# Species-specific lipophilicities of fluorinated diketones in complex equilibria systems and their potential as multifaceted reversible covalent warheads

**DOI:** 10.1038/s42004-023-01004-2

**Published:** 2023-09-15

**Authors:** Ishay Columbus, Lee Ghindes-Azaria, Ido Michael Herzog, Eliav Blum, Galit Parvari, Yoav Eichen, Yoram Cohen, Eytan Gershonov, Eyal Drug, Sigal Saphier, Shlomi Elias, Boris Smolkin, Yossi Zafrani

**Affiliations:** 1 Department of Organic Chemistry, Israel Institute for Biological Research, Ness Ziona, Israel; 2 Schulich Faculty of Chemistry, Technion − Israel Institute of Technology, Technion City, Haifa, Israel; 3 School of Chemistry, Tel Aviv University, Tel Aviv, Israel; 4 Department of Analytic Chemistry, Israel Institute for Biological Research, Ness Ziona, Israel

**Keywords:** Structure elucidation, Drug discovery and development, Chemical tools, Target identification

## Abstract

Combined molecular, physicochemical and chemical properties of electrophilic warheads can be applied to create covalent drugs with diverse facets. Here we study these properties in fluorinated diketones (FDKs) and their multicomponent equilibrium systems in the presence of protic nucleophiles, revealing the potential of the CF_2_(CO)_2_ group to act as a multifaceted warhead for reversible covalent drugs. The equilibria compositions of various FDKs in water/octanol contain up to nine species. A simultaneous direct species-specific ^19^F-NMR-based log *P* determination of these complex equilibria systems was achieved and revealed in some cases lipophilic to hydrophilic shifts, indicating possible adaptation to different environments. This was also demonstrated in ^19^F-MAS-NMR-based water-membrane partitioning measurements. An interpretation of the results is suggested by the aid of a DFT study and ^19^F-DOSY-NMR spectroscopy. In dilute solutions, a model FDK reacted with protected cysteine to form two hemi-thioketal regioisomers, indicating possible flexible regio-reactivity of CF_2_(CO)_2_ warheads toward cysteine residues.

## Abstract (web summary)

Fluorinated ketones can function as covalent warheads applied in the design of reversible covalent inhibitors, however, the reactivity of certain fluorinated moieties remains underexplored. Here, the authors investigate the species-specific lipophilicities of fluorinated geminal diketones in multicomponent equilibrium systems and reveal their potential as multifaceted warheads for reversible covalent drugs.

## Introduction

**Fig. 1:**
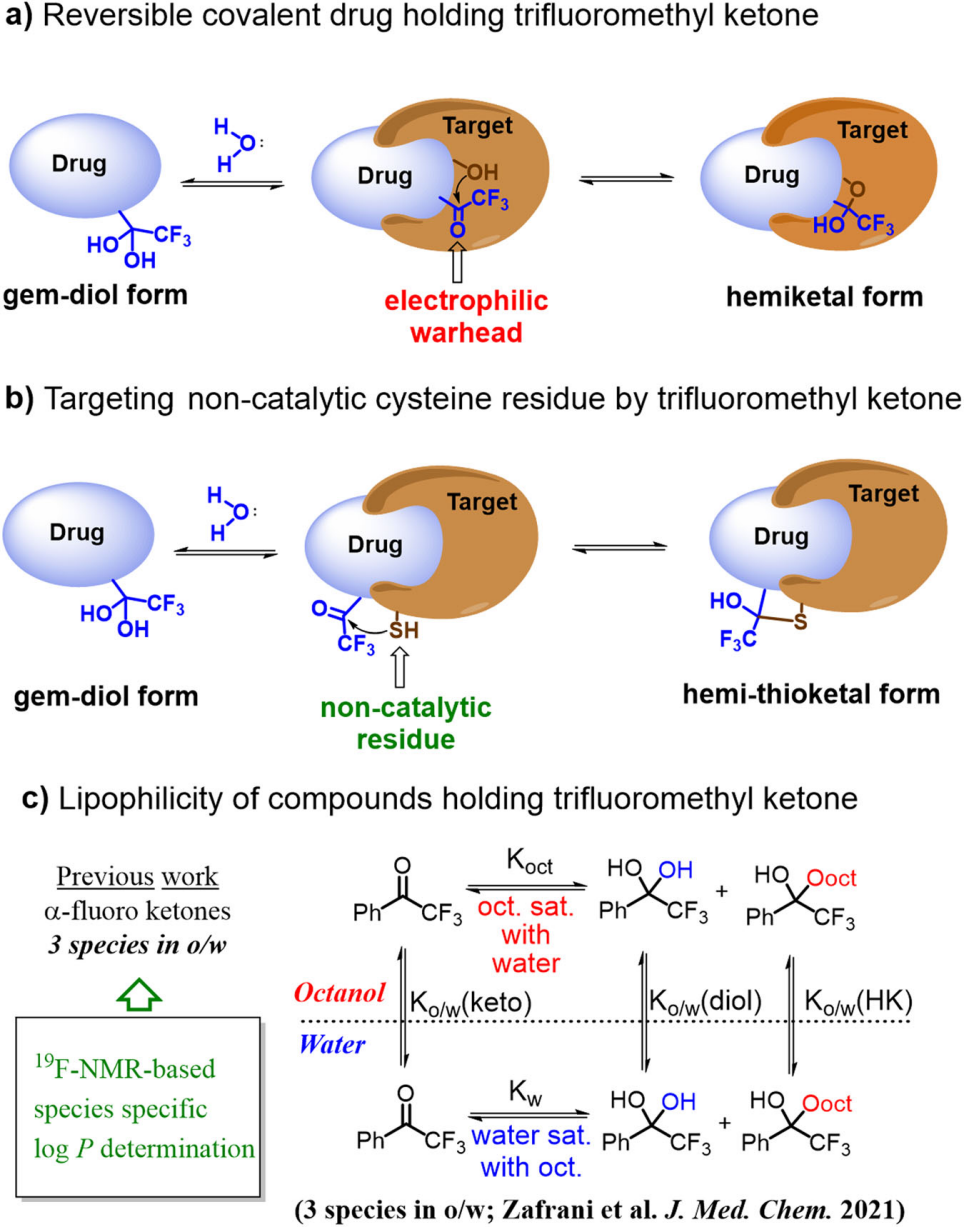
The trifluoromethyl ketone warhead. **a** Equilibrium of trifluoromethyl ketones in aqueous solutions and reaction in the catalytic active site of a target protein. **b** Reaction in a non-catalytic site of a target protein. **c** Equilibria scheme of log *P* determination of trifluoromethyl acetophenone.

The rational design of covalent drugs has drawn increased attention in recent years^1,2^, mainly due to the shift from covalent inhibitors that exploit the catalytic machinery of enzymes to targeting non-catalytic residues^3^. In particular, reversible covalent inhibitors show great promise since irreversible bonding may potentially increase toxicity via undesired off-target reactions and protein modifications that could lead to an immune response^4,5^. A compelling example of reversible inhibitors are compounds that hold the di- and trifluoromethyl ketone functions, which are able to form reversible covalent bonds mainly at enzymatic catalytic sites via hemiketal formation (Fig. 1a)^6,7^. Many of the biologically active compounds that hold the α-fluorinated ketone moiety, acting as an electrophilic warhead, are short peptides derivatives^8^. An interesting feature of α-fluorinated ketones is that in aqueous solutions they exist in equilibrium with their hydrated forms (gem diol). Bioactive compounds holding a trifluoromethyl ketone function were shown to act as reversible covalent inhibitors of acetylcholine esterase^9^, phospholipases^10^, dengue NS2B-NS3pro serine protease^11^ and more^12^. The α-difluorinated carbonyl function has also been incorporated into various structures, including peptides, which have been used as inhibitors for different proteases^13–15^ with a recent example being a possible inhibitor against cysteine SARS-Cov-2 main protease^16^. However, in all of these examples, the electrophilic warhead reacts at the catalytic site (Fig. 1a), while making use of fluorinated ketones for targeting non-catalytic amino acid residues, for instance cysteine, is extremely rare (Fig. 1b). An example of such an application has very recently been reported by Ding and co-workers who targeted a non-catalytic cysteine residue with an aromatic trifluoromethyl ketone warhead in the design of a reversible covalent kinase inhibitor^17^. As mentioned above, this is a promising new strategy to obtain stronger and more specific binding of drugs. Such a design of modified and novel lead compounds requires a structure-guided design considering the location of cysteine residue(s) relative to the drug’s target site. Note that due to the high nucleophilicity of the thiol group and relatively low abundance in proteins, the cysteine residue is considered the ideal nucleophile for non-catalytic targeted covalent inhibitors with high levels of specificity and prolonged duration of action^18,19^.

Small molecule drug design requires balancing target site specificity with essential physicochemical properties. Appending a covalent electrophilic warhead to an existing lead/drug may lead to changes in physicochemical properties that the attached group causes which may in turn affect the compound’s pharmacokinetic properties. Therefore, the expected influence of the various electrophilic warheads on the physicochemical properties of the drug molecules, e.g., on the lipophilicity, polarity, H-bond ability and more, is of great importance. Lipophilicity in particular, is a key property as it dictates drug disposition in the body (ADME), influencing both efficacy and toxicity^20^. In recent years, there has been a growing focus in the medicinal chemistry arena on systematically studying the lipophilicity (log *P*) changes obtained by designing small molecules, in particular while studying bioisosteres^21^, and investigating their structure-lipophilicity-relationship, for example, the effects different fluorination patterns have on lipophilicity^22–29^. However, surprisingly, such discussion seems absent from the literature dealing with the design of targeted covalent inhibitors. Moreover, studying the lipophilicity of compounds containing warheads that can bind reversibly and may consist of several forms in equilibrium, further complicates the situation and is highly challenging. Recently, we have shown for the first time, that a direct measurement of species-specific log *P* values of α- di- and trifluoromethyl phenyl ketones and their hydrated forms, could be determined using Linclau’s^30^ ^19^F-NMR log *P* method (Fig. 1c)^31^. Thus, this method paved the way for the determination of species-specific log *P* values of any drug or bioactive compound holding fluorinated ketone functions as warheads.

### Multifaceted reversible covalent warhead—the new proposed concept

**Fig. 2:**
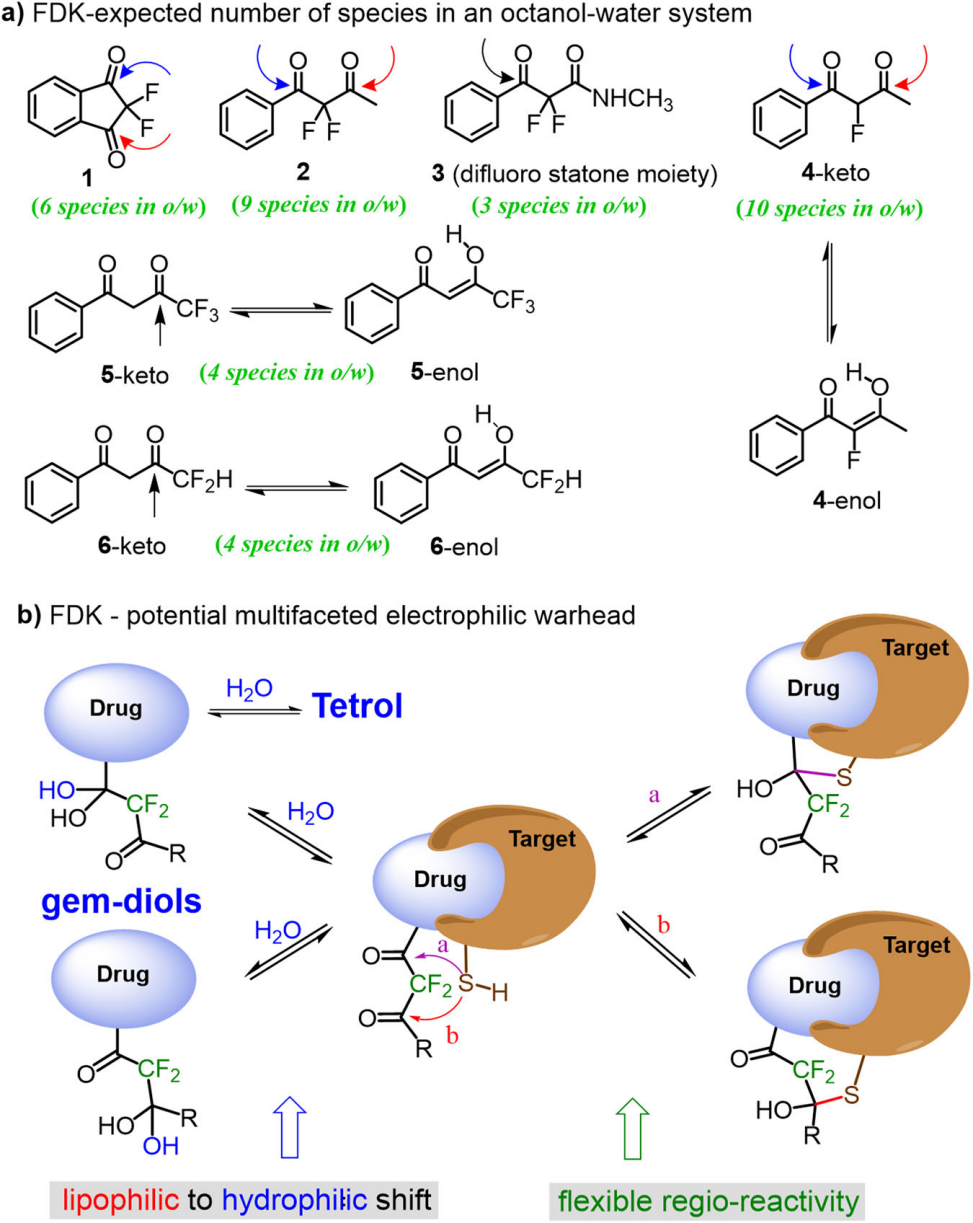
The potential of FDKs as electrophilic warheads. **a** Points of nucleophilic attack and the expected number of species in octanol-water phases for compounds **1-6**. **b** The concept of multifaceted reversible covalent warheads.

Adding a second carbonyl group to the α-fluorinated keto moiety, such as in the fluorinated geminal diketones (FDK) presented in Fig. 2a may lead to structures with growing complexity and interesting properties. The known difluorostatone moiety (fluorinated β-keto amide as in compound **3**) has been used as a building block for the design of specific inhibitors for many enzymes including Human leukocyte elastase^32^, HIV-1 protease^33^ and malarial PfSUB1 protease^34^. However, in structures holding this electrophilic warhead only one carbonyl is active toward a nucleophilic attack. On the other hand, an additional active carbonyl, such as in compounds **1,** **2** and **4**, will lead to a gem-fluorinated dicarbonyl analogue, i.e. CF_n_(C = O)_2_, holding a 1,3-dielectrophilic center which may potentially act as a unique and flexible warhead (Fig. 2a). As this function consists of two independent carbonyl electrophiles, we envisioned that it may pose (a) the flexibility to react with either electrophilic centers, and (b) the ability to impart a wide range of log *P* values (hydrophilic to lipophilic^35^) by its different forms under physiological conditions, a combination of features which led us to term this function a potential multifaceted reversible warhead (Fig. 2b). To examine the concept of the CF_n_(C = O)_2_ function as a potentially multifaceted warhead, we initially studied the complex equilibria compositions expected from FDK **1-6** model compounds in octanol-water phases followed by measuring the species-specific lipophilicities of the multiple components obtained, and then investigating the regio-reactivity of selected examples with nucleophilic amino acids such as cysteine.

Characterizing the equilibria systems of compounds **1-6** in octanol-water and determining the species-specific log *P* value of each component is highly challenging, since compounds such as diketones **1,** **2** and **4**, will have multiple species (six, nine and ten species, respectively) at equilibrium (Fig. 2a). We show that the specific log *P* values of most of the species involved, i.e. ketones, enols and hydrated forms, can be reliably determined directly and simultaneously by the ^19^F-NMR-based method. The wide range of lipophilicities obtained, in some cases in an equilibrium system that holds both lipophilic and hydrophilic species, indicates the potential compatibility to different physiological environments, as we also demonstrate using water-membrane partitioning measurments with FDK **1** as a model. We also suggest an interpretation of the results aided by a DFT study and by ^19^F-diffusion ordered spectroscopy (DOSY)-NMR which was performed for studying both signal assignment and species-solvent interactions (H-bonding).In the final section, we show in a series of reactions that FDK **2** as an asymmetric model, reacts with a protected cysteine at both carbonyls, forming the corresponding hemi thioketal regioisomers, emphasaizing the potential of compounds holding a CF_2_(CO)_2_ warhead to bind to proteins via a non-catalytic cysteine residue with high flexibility.

## Results and discussion

The varied FDKs **1-6** described here, demonstrating potential structures for multifaceted warheads, were designed to systematically study the effects of various fluorination patterns on their chemical, molecular and physicochemical properties. Compounds **1-4** were prepared using Selectfluor as described by Tang et al.^36^, while compounds **5** and **6** are commercially available. When distributing multicomponent species between octanol and water (or buffer), equilibrium is obtained for each species according to their own molecular properties and tendency to partition between the two phases. Having more than one species at equilibrium requires elucidation of the micro or specific-log *P* for each species separately. This can be done successfully and directly by the ^19^F-NMR-based method as recently independently demonstrated for much simpler systems by us^31^ (see Fig. 1b) and by Linclau et al.^37^.

### Species identification using NMR spectroscopy

For the determination of the specific log *P* value of each species in the equilibria systems, each ^19^F-NMR signal had to be assigned first to a particular species in both water and octanol saturated with water, separately. For all systems, both ^19^F- and ^13^C-NMR measurements were used to identify the species involved in the equilibria studied, and then ^19^F-NMR was used for the species-specific log *P* determination. Note that for characterization of multicomponent equilibrium systems, NMR spectroscopy has exclusive advantages over other methods, since other common analytical tools (GC, HPLC, MS, UV and IR) are not useful for this purpose^38^. Full details of the results and the analyses of the equilibria systems of DFKs **1-6** are presented in Supplementary Note 1 (Table S1) and in Supplementary Data 2. Since species distribution in water is most relevant to in-vivo physiological conditions, the equilibrium composition in aqueous solutions of each of the compounds **1-6** was first studied in water. This also proved to be helpful, since in water only hydrates and no octyl hemiketals could be formed, making the initial species identification easier. In view of the differences in the chemical shifts between ^19^F-NMR signals of the various species observed, good signal separation was obtained in most cases. The assignment starting point in water was based on an upfield shift of the hydrated forms relative to the ketones as previously observed^31^. Moving to the more complex ^19^F-NMR spectra in octanol saturated with water, the hemiketal forms of compounds **1**-**3** and **6**, are characterized by an AB quartet (ABq) system owing to the diastereotopic nature of the fluorine atoms, a phenomenon that further facilitated signal assignment. For similar species in the equilibrium, i.e. regioisomers of gem-diols or hemiketals, where differentiation by ^19^F-NMR was not possible, ^13^C-NMR was used to determine the correct assignment for each of those species, according to known chemical shifts and prevalence in the solution (see Supplementary Data 2).

**Fig. 3:**
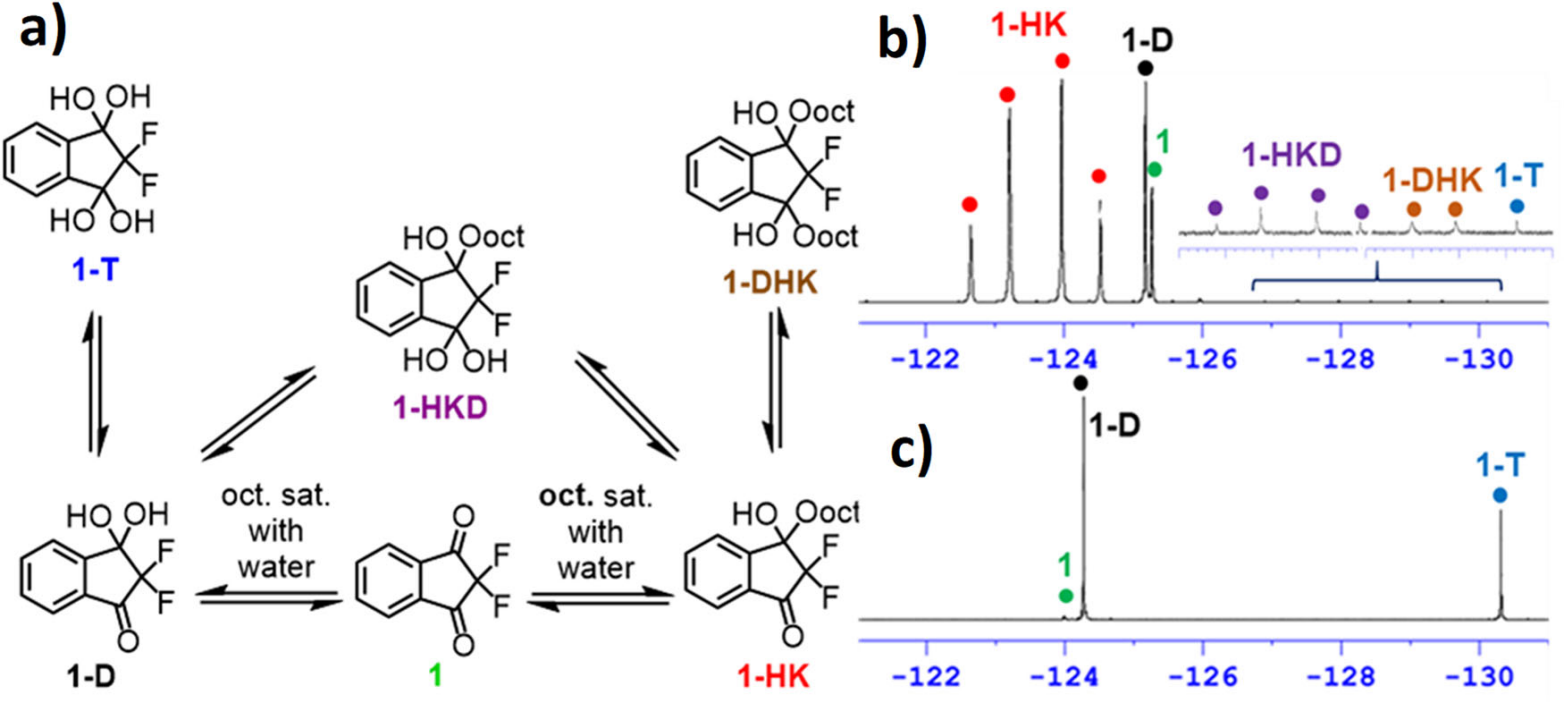
Species observed for FDK 1 in octanol saturated with water. **a** Distribution of species: **1** (7.1%), **1-HK** (74.5%), **1-D** (17.6%), **1-T** (0.06%), **1-HKD** (0.42%) and **1-DHK** (0.26%). **b** ^19^F-NMR spectrum of **1** in octanol saturated with water, and **c** spectrum of **1** in water. Inspection of the spectra reveals that no peak overlapping was observed, indicating the high potential for species-specific log *P* determination for those species that appear in both phases. The minor differences in the chemical shifts of the signals may be attributed to the solvent effect.

For the equilibrium composition study in each medium, we began with the relatively simple symmetric diketone **1**. In the octanol-water system it may exist in up to six species in equilibrium including diketone **1**, keto-diol **1-D**, tetrol **1-T**, hemiketal **1-HK**, hemiketaldiol **1-HKD** and di-hemiketal **1-DHK**, as shown in Fig. 3. In the aqueous solution of diketone **1**, only three species were expected to exist in equilibrium, and indeed **1,** **1-D** and **1-T** were all observed by ^19^F- and ^13^C-NMR (Figs. 3c and S1, S2 with added detailed explanation, Supplementary Data 2). Owing to the high electronegativity of the α-fluorine atoms as well as the added carbonyl (electron withdrawing group, EWG), both carbonyls act as electrophiles, and therefore the hydrated forms are the major species in water, with diketone **1** comprising less than 2% of the molecular population. Next, **1** was dissolved in octanol saturated with water, emulating the phase which will be formed in the following log *P* determination experiment. Under these conditions, both hydrated and hemiketal forms are expected to be observed, and indeed, all six species, i.e. compounds **1,** **1-D,** **1-T,** **1-HK,** **1-HKD** and **1-DHK**, were detected and identified by ^19^F- and ^13^C-NMR (Figs. 3b and S1, S2, Supplementary Data 2).

**Fig. 4:**
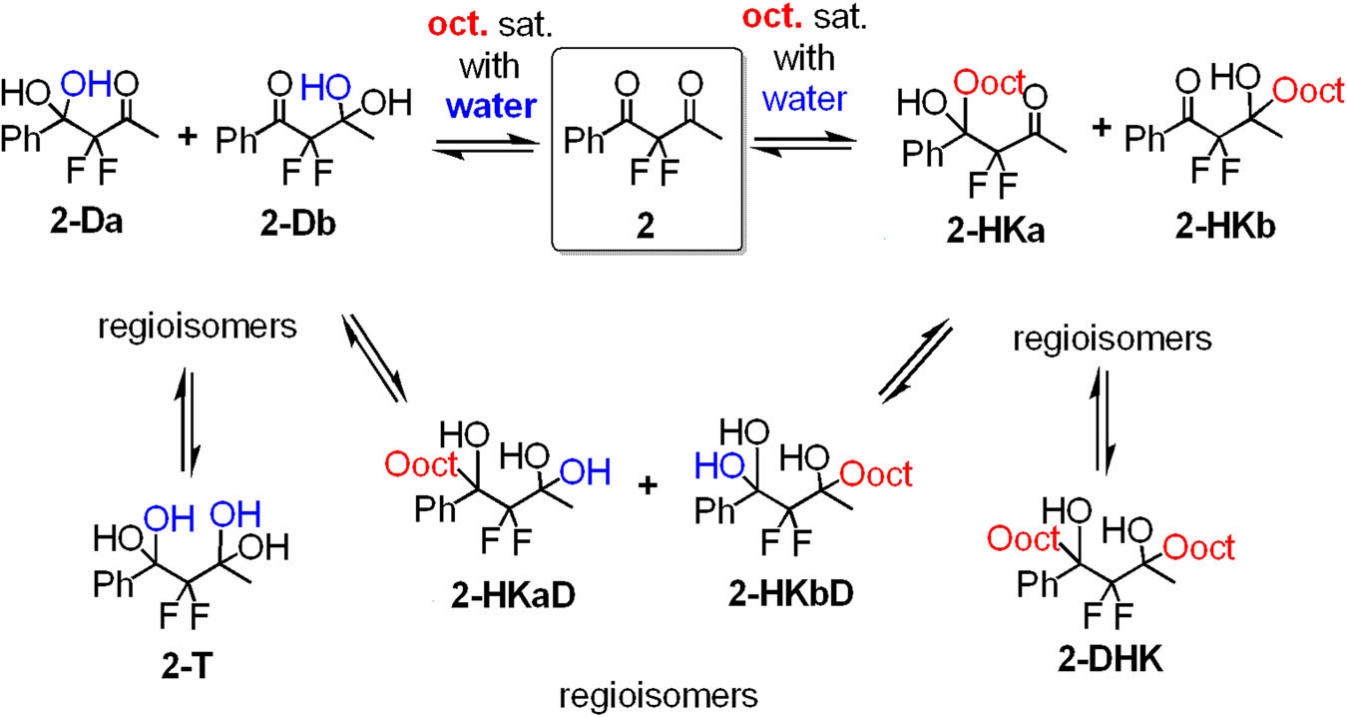
Species observed for FDK 2 in octanol saturated with water. Distribution of species: **2** (22.7%), **2-HKa** (20.9%), **2-HKb** (21.2%), **2-DHK** (0.38%), **2-HKaD** (2.9%), **2-HKbD** (2.0%), **2-Da** (4.8%), **2-Db** (24.5%) and **2-T** (0.57%).

A bigger challenge for analysis was the asymmetric diketone **2**, for which as many as nine species may exist in equilibrium in the octanol-water biphasic system (Fig. 4). The identification of all species, mainly the corresponding regioisomers, was facilitated by ^19^F- and ^13^C-NMR and comparison to reference compounds (for details see Supplementary Data 2). Moreover, for this complex equilibrium system we used ^19^F-DOSY-NMR spectroscopy as a powerful tool, not only for strengthening the assignment of the species signals, but also to obtain useful information about their interactions, mainly H-bonding with the protic solvents (vide infra). In the aqueous solution of **2**, all possible hydrated forms were observed by ^19^F- and ^13^C-NMR analysis, i.e. **2,** **2-Da,** **2-Db** and **2-T** (Figs. S4, S5, Supplementary Data 2). Again, the population of diketone **2** (2.1%) in this equilibrium is much smaller than that of its hydrated forms, mainly the diol species, indicating the expected behavior of drugs holding this moiety under in-vivo physiological conditions. Moreover, in octanol saturated with water, all nine species were observed and could be identified by ^19^F-NMR spectroscopy (Figs. 4 and S4, Supplementary Data 2).

**Fig. 5:**
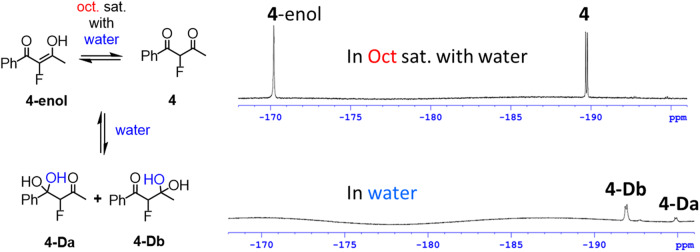
Species observed for FDK 4. Distribution of species in water: **4-Da** (19.1%), **4-Db** (80.9%). Distribution of species in octanol saturated with water: **4-enol** (51.7%), **4** (48.3%).

In addition to the diketones **1** and **2**, difluorostatone **3** was prepared due to its importance for medicinal chemistry and for comparison purposes. For this compound, as expected, only one carbonyl was found to be active towards oxygen nucleophiles in the above-mentioned conditions, i.e. the keto moiety alone and not the amide moiety (Fig. S6, Supplementary Data 2). Monofluoro**-4**, trifluoromethyl-**5** and difluoromethyl-**6** were designed so that, in addition to the hydrates and hemiketals formed, enols may also be formed to delve into this added complexity. Interestingly, when the monofluoro diketone **4** was dissolved in water, only the hydrated forms **4-Da** and **4-Db** were observed. In octanol saturated with water the monofluoro diketone **4** appears together with its tautomer **4-enol**, however, no hemiketals nor hydrated species were observed to any significant amount (only non-definite traces) under these conditions (Figs. 5 and S9, Supplementary Data 2). This analysis was supported by both ^1^H- and ^19^F-NMR spectroscopy of **4** in the deuterated solvent CDCl_3_ (Fig. S8, Supplementary Data 2). Since no common species were observed in the two phases (Fig. S10, Supplementary Data 2), we could not measure the log *P* values of the species involved in the solutions of **4**. However, the formation of the hydrated forms **4**-**Da** and **4**-**Db** in water (and the suspected trace hemiketal species in octanol), stands in contrast to what we have previously observed under similar conditions for the related α-monofluoro mono ketone which is not reactive enough to form a diol or hemiketal with water or octanol, respectively^31^. This implies that the monofluoro gem-diketo moiety CHF(C = O)_2_ may also act as a potential electrophilic warhead for reversible covalent inhibition.

When placing a fluorine moiety, either trifluoromethyl (**5)** or difluoromethyl (**6**), α to one carbonyl but γ to the other, only the carbonyl adjacent to the fluorine atoms becomes activated and a “regular” warhead is formed. For diketones **5** and **6**, the equilibrium in octanol-water system was more balanced than that observed for diketone **4** and enabled us to determine log *P* for the enol forms (Figs. S11–S16, Supplementary Data 2).

### Species-specific log *P* determination

Having identified each species in the above-described multicomponent equilibrium systems, we could now turn to perform the species-specific log *P* measurements. As mentioned above, the ^19^F-NMR-based method was used for the specific log *P* determination^30,31^. The population of compound X in each phase is determined by integrating its signals against a reference compound (trifluoroethanol, appearing at −77.1 ppm) having a known log *P* value. The log *P* of compound X is calculated by eq. (1), where I is the integration value of the relevant signal.

1 $${\log {P}_{x}}_{o/w}=\log {P}_{{ref}}+\log \left(\frac{{{I}_{x}}_{o}}{{{I}_{x}}_{w}}* \frac{{{I}_{{ref}}}_{w}}{{{I}_{{ref}}}_{o}}\right)$$

Note that using this method, systematic errors are eliminated due to the compensation effect inherent to the determination of a ratio of a ratio, and therefore, there is also no need to weigh the exact amount of the compound or measure the phase volume. In addition, only discreet signals corresponding to the measured compound are integrated (Ix), and thus impurities, if no interactions occur, do not affect the results. The phases separated during the log *P* “stir flask” experiments contain different concentrations of each species according to their partition coefficient, and therefore, outside the range −4 to 4 (8 log *P* units), the concentration of the compounds in one of the phases may be below the limits of detection.

**Fig. 6:**
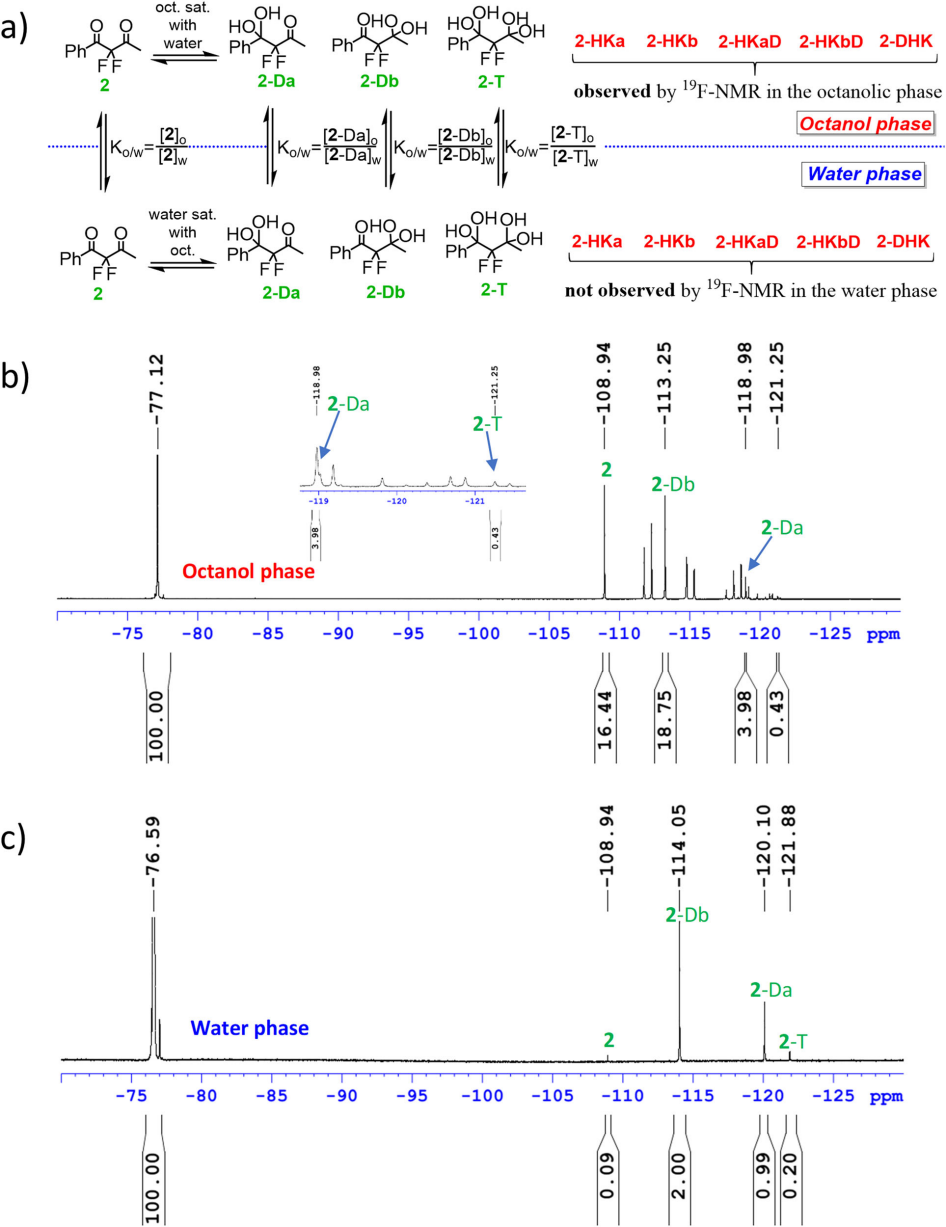
Log *P* measurement of FDK 2 based on the ^19^F-NMR method. **a** The species involved in the equilibria in both phases. **b** ^19^F-NMR spectrum of **2** in the octanol phase, and **c** spectrum of **2** in the water phase, after a stir-flask experiment.

**Fig. 7:**
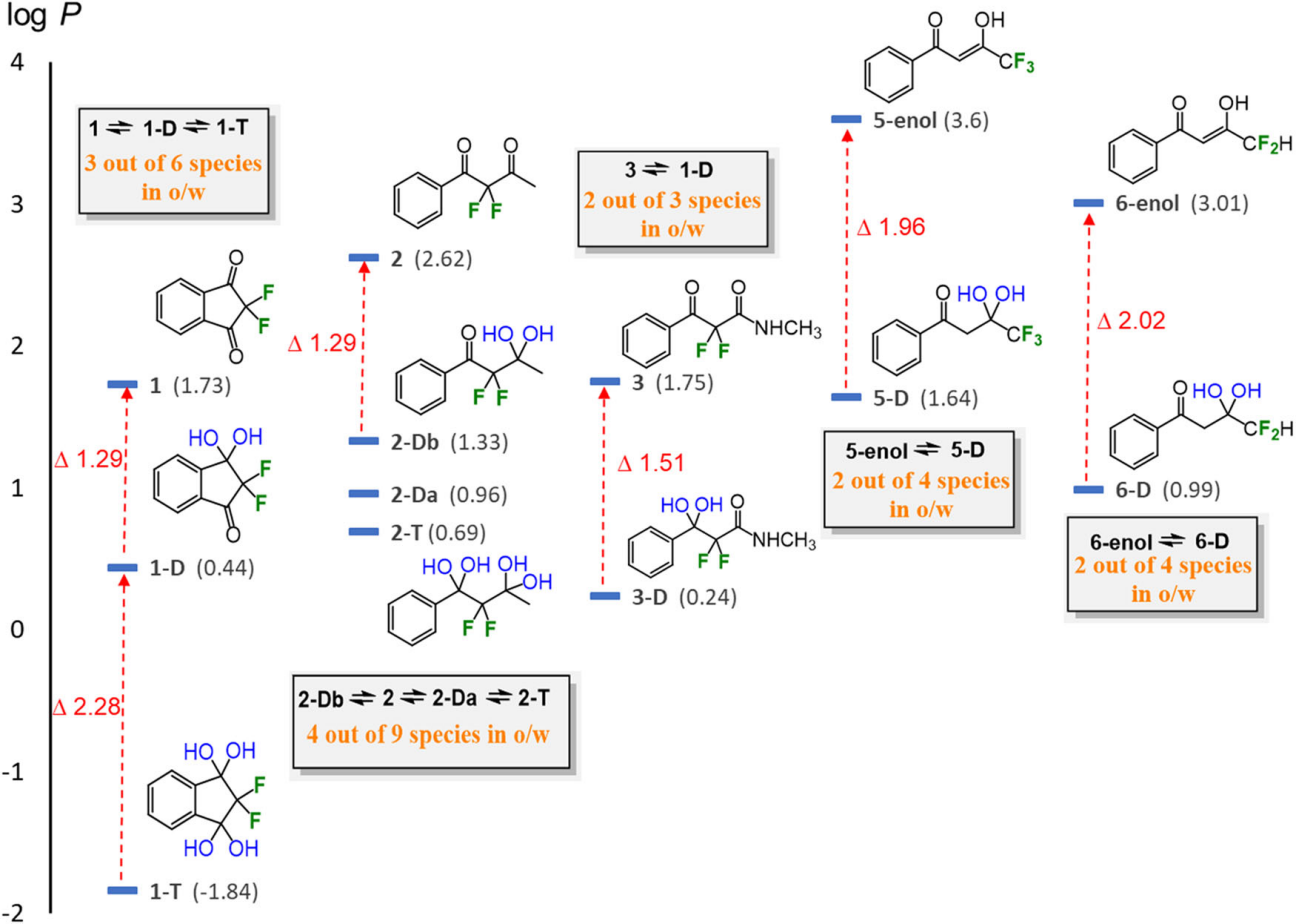
Lipophilicities of FDKs 1-6 and their hydrated forms. Log *P* values of the various diketones and their species in equilibrium measured using the ^19^F-NMR method.

Figure 6 illustrates the most complex example of the ^19^F-NMR-based species-specific log *P* determination in equilibria systems, that of compound **2**. Four species, i.e. **2,** **2-Da,** **2-Db** and **2-T** were observed in both octanol and water phases, and hence, their specific log *P* values could be extracted (vide infra Fig. 7). The most lipophilic species observed in both phases is diketone **2**. It is easy to observe that most of **2** partitioned to the octanolic phase (Fig. 6b), while the signal of **2** is quite small in the water phase (Fig. 6c). More lipophilic species, i.e. the hemiketals **2-HKa,** **2-HKb,** **2-HKaD,** **2-HKbD** and **2-DHK** were not observed in the water phase. A very small amount of these ketals may have partitioned to the water phase, however, their concentrations were below the ^19^F-NMR limit of detection (log *P* > 4). An additional limitation may arise when one of the species in equilibrium exists in minute amounts. For example, the tetrol **2-T** is only 5.6% in water and 0.57% in octanol (Table S1, Supplementary Information), however, in this case even at these low levels, we were able to determine its log *P* value. For the water-soluble species, **2-Da** (deconvolution was required) and **2-Db**, large signals were recorded in both spectra, and their log *P* values were easily determined. The species-specific log *P* values from all equilibria systems described above for FDKs **1**-**3,** **5-6** are graphically presented in Fig. 7. Similarly to the above-mentioned analysis of diketone **2**, the simpler examples of the symmetric diketone **1**, difluorostatone derivative **3**, and diketone-enoles **5** and **6** were analyzed, and the specific log *P* values of the species that appeared in both phases after separation were successfully determined (Figs. 7 and S3, S7, S13 and S16, Supplementary Data 2).

Looking at the measured log *P* values, it was found that the symmetric diketone **1** exhibits a log *P* value of 1.73, while as expected, its gem-diol form **1-D** exhibits a more hydrophilic nature, lowering the lipophilicity by more than one log *P* unit relative to diketone **1**. Hydration of the second carbonyl (**1-T**) gave an even more dramatic effect of more than two log *P* units, leading to a hydrophilic log *P* value of −1.84. A drug having such lipophilic-hydrophilic “flexibility” (multifaceted), may dissolve in aqueous environments, such as in the intestinal lumen, yet be able to cross membranes and be absorbed via equilibrium with its more lipophilic form, and later enter hydrophobic binding pockets in the lipophilic keto form to react as an electrophilic warhead. Similar to conformational adaptors described by Müller and co-workers^39^, compounds holding the warhead CF_2_(C = O)_2_ may also be considered as adaptors, which change their physicochemical properties according to their chemical/biological environment. Interestingly, diketones **2** and **3** showed a similar Δlog *P* for the first hydration exchange, i.e. to **2-Da/2-Db** and **3-D**, respectively. This shows the potential for generality of the effect and may be used as a tool when planning novel structures, where a Δlog *P* of about 1.3 may be expected for keto-diol transition. However, it was found that tetrol **2-T** did not show a dramatic decrease in lipophilicity as its rigid counterpart **1-T** has shown, and even compared to its diol counterparts **2-Da** and **2-Db**. We assume that this may be due to the existence of two strong intramolecular H-bonds between both the 1,3 dihydroxyls, which may cause a significant enhancement of the lipophilicity, via reducing the H-bond interactions with the solvents. Such a double intramolecular H-bond may exist in tetrol **2-T**, as implied by the ^19^F-DOSY-NMR analysis described below, while only one such interaction is possible in tetrol **1-T**, as we indeed show by the DFT study in the next section (vide supra). In order to evaluate the relevance of the measured lipophilicities to biological systems, the species-specific lipophilicities of **2** were also measured at physiological temperature, 37 ^o^C, and the log *P* values of the species **2,** **2-Da,** **2-Db** and **2-T**, were found to be quite similar to those measured at room temperature (log *P* 2.70, 1.28, 0.88 and 0.65, respectively, see Fig. 7 for the values at RT).

For the terminally fluorinated FDKs **5** and **6**, as mentioned above, the diketone forms were not observed in both phases after separation. However, to our satisfaction the enol forms were observed in both phases and exhibited relatively high lipophilicities. This indicates an intramolecular H-bond between the ketone and the γ-enol hydroxyl, which is a well-known phenomenon. These intramolecular H-bonds together with the expected reduction in both H-bond basicity and polarity, cause an increase in lipophilicity. For both structures, the keto-diol forms **5-D** and **6-D** show a large, two-unit reduction in log *P* relative to the enol forms **5-enol** and **6-enol**, respectively.

Using accessible software programs to calculate theoretical log *P* such as Clog *P* or Milog *P* to predict the lipophilicities of these forms lead to unreliable predictions with gaps in predictions, sometimes even larger than 1.5 log *P* units for the tetrol forms (See Table S2, Supplementary Note 2). This emphasizes the importance of this method which gives access to knowledge of the true nature of these species.

### ^19^F-DOSY-NMR spectroscopy for the species identification in equilibrium

**Fig. 8:**
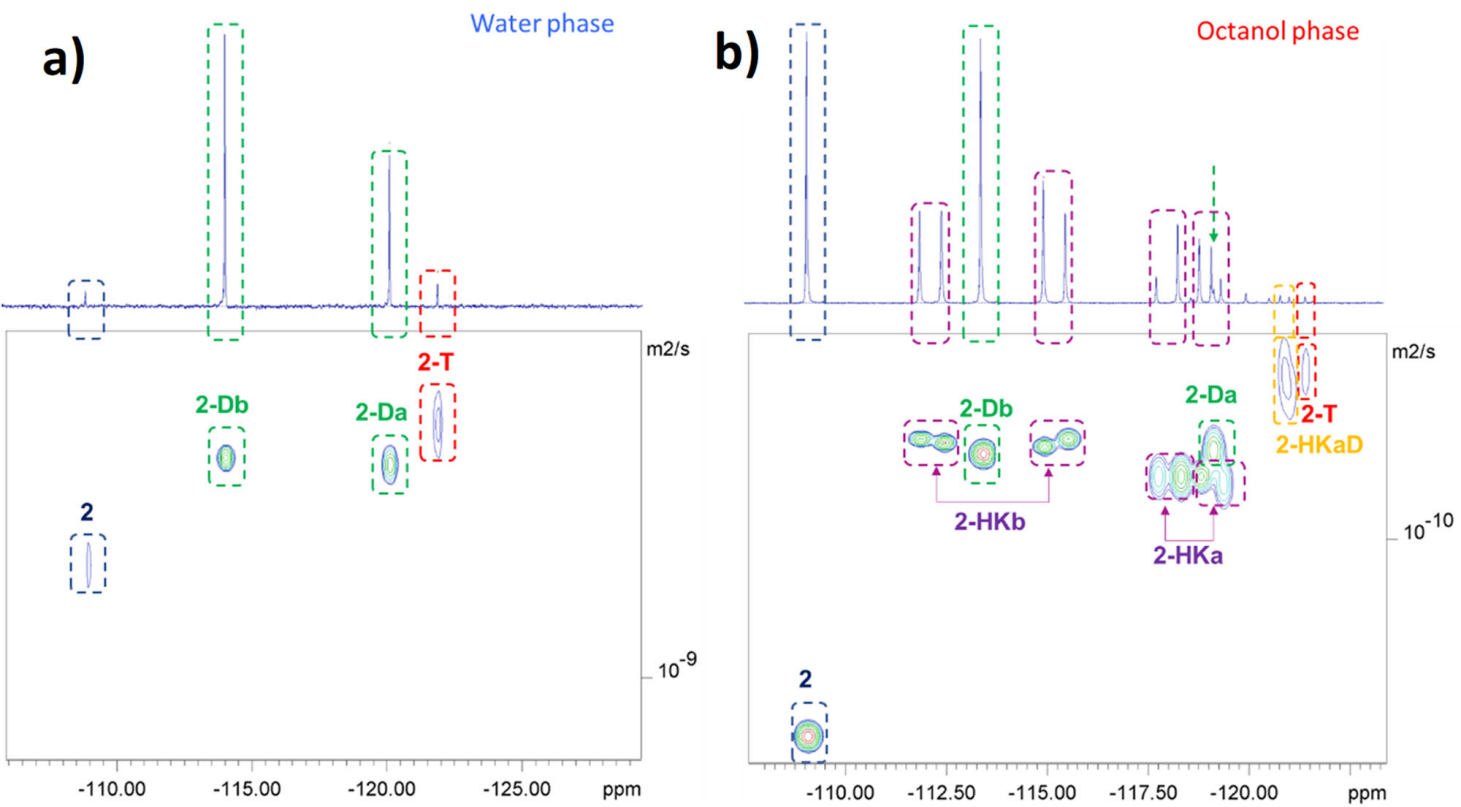
^19^F-DOSY-NMR of FDK 2. **a** Spectra of the equilibria mixtures obtained in water, and **b** in octanol, in the log *P* determination experiment of **2**.

**Fig. 9:**
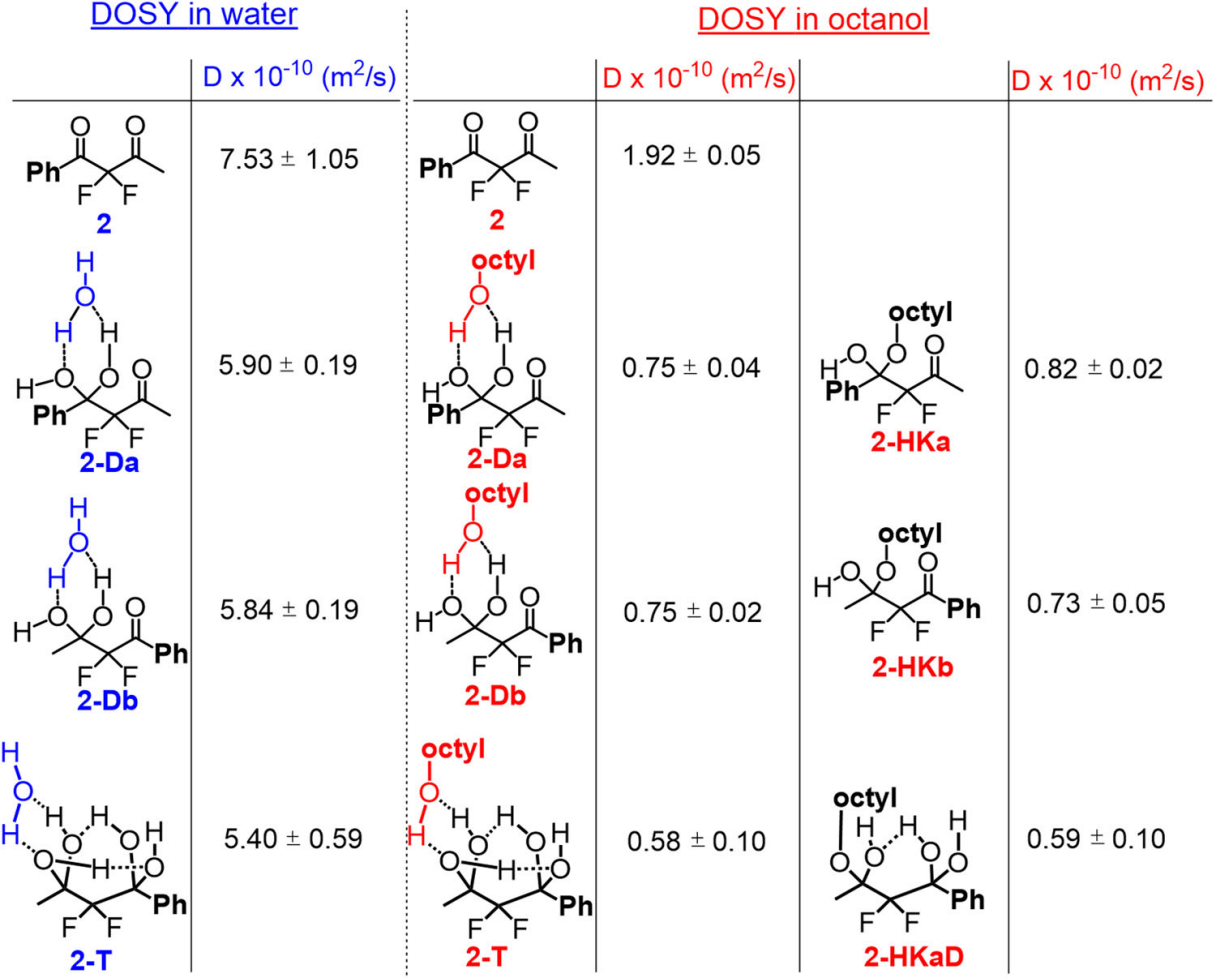
Diffusion coefficients of FDK 2. The diffusion coefficients of the proposed species involved in the log *P* determination of **2**. The magnitude of the errors is inversely proportional to the concentrations of the species.

To further characterize the most complex equilibrium systems of **2** and **1** and reinforce our findings and interpretations about the differences between these acyclic and cyclic FDKs, respectively, we used the ^19^F-DOSY-NMR spectroscopy technique. This powerful tool is traditionally used for the characterization of NMR spectra of organic mixtures and is based on the correlation between the diffusion coefficient of a given structure and its molecular weight. In the present study we assumed that this analytical technique may be applied in an unusual way, to support the characterization of the equilibrium system of the asymmetric FDK **2**, in each separate phase. In addition, we assumed that useful information can be obtained not only for the assignment of the species signals but also for their interactions with the solvents. Indeed, the spectra presented in Fig. 7 clearly provide this information, which may also help in the interpretation of the findings relating to the lipophilicity trends discussed above. The ^19^F-DOSY-NMR spectra were studied using the standard eddy currents delays (LED) pulse sequence. Starting with the simpler spectrum, i.e. the four-component equilibrium system in water (Fig. 8, left), we observe that the D value (diffusion coefficient) of diketone **2** is the highest one, while the tetrol **2-T**, obtained from the covalent addition of two water molecules, has the lowest D value (see also Fig. 9). These diffusion coefficients correlate as expected with the increase in molecular weight and therefore strengthen our signals assignment. The regioisomeric gem-diol species **2-Da** and **2-Db**, formed from the addition of one water molecule to diketone **2**, exhibit as expected, very close D values to each other. We assume that the non-linear reduction in the D values of these four components may be related to the inter-molecular H-bonding with water molecules as suggested in Fig. 9. The gem-diol function is more prone than carbonyl to interact with a water molecule, as both H-bond acceptor and donor, to form a bimolecular H-bonded 6-membered ring (each gem-diol group can bind at most two water molecules). The relatively smaller decrease in the D value of tetrol **2-T** implies that this 1,3-di-gem-diol may additionally be involved in intramolecular H-bonds, which limits its interactions with water molecules, a motif that is well-known to increase lipophilicity (vide infra).

Moving to the more viscous octanolic phase, all D values of the four components **2,** **2-Da,** **2-Db** and **2-T** were reduced appropriately (Figs. 8, 9). However, while the reduction in the diffusion coefficient of diketone form **2** can be explained by the increased viscosity from water to octanol, the more pronounced reduction in the D values of the hydrated forms **2-Da,** **2-Db** and **2-T**, may be attributed to a strong H-bond interaction between the gem-diol moiety and an octanol molecule. The diffusion coefficients of hemiketals **2-HKa** and **2-HKb** in octanol were found to be quite similar to those of diols **2-Da** and **2-Db**, reinforcing this suggestion of involvement of H-bond interactions of the gem-diol group with the solvent (octanol). Note that for the analysis and signal integration, this diffusion measurement analysis strengthens our peak assignments, mainly in cases such as **2-Da**, for which a deconvolution of its signal from that of hemiketal **2-HKa** was required for its ^19^F-NMR-based log *P* determination. Thus, we conclude that the signal assignments of FDK **2** and its hydrated and hemiketal forms in the octanol-water system is well established. For similar findings and interpretation by ^19^F-DOSY-NMR for FDK **1**, see Supplementary Note 3 (Table S3). As far as we are aware this is the first time that DOSY-NMR technique is used to not only for the substantiation of signal assignment of such multicomponent equilibrium systems, but also in the exploration of inter and intramolecular hydrogen bonding with the species involved.

### DFT study

**Fig. 10:**
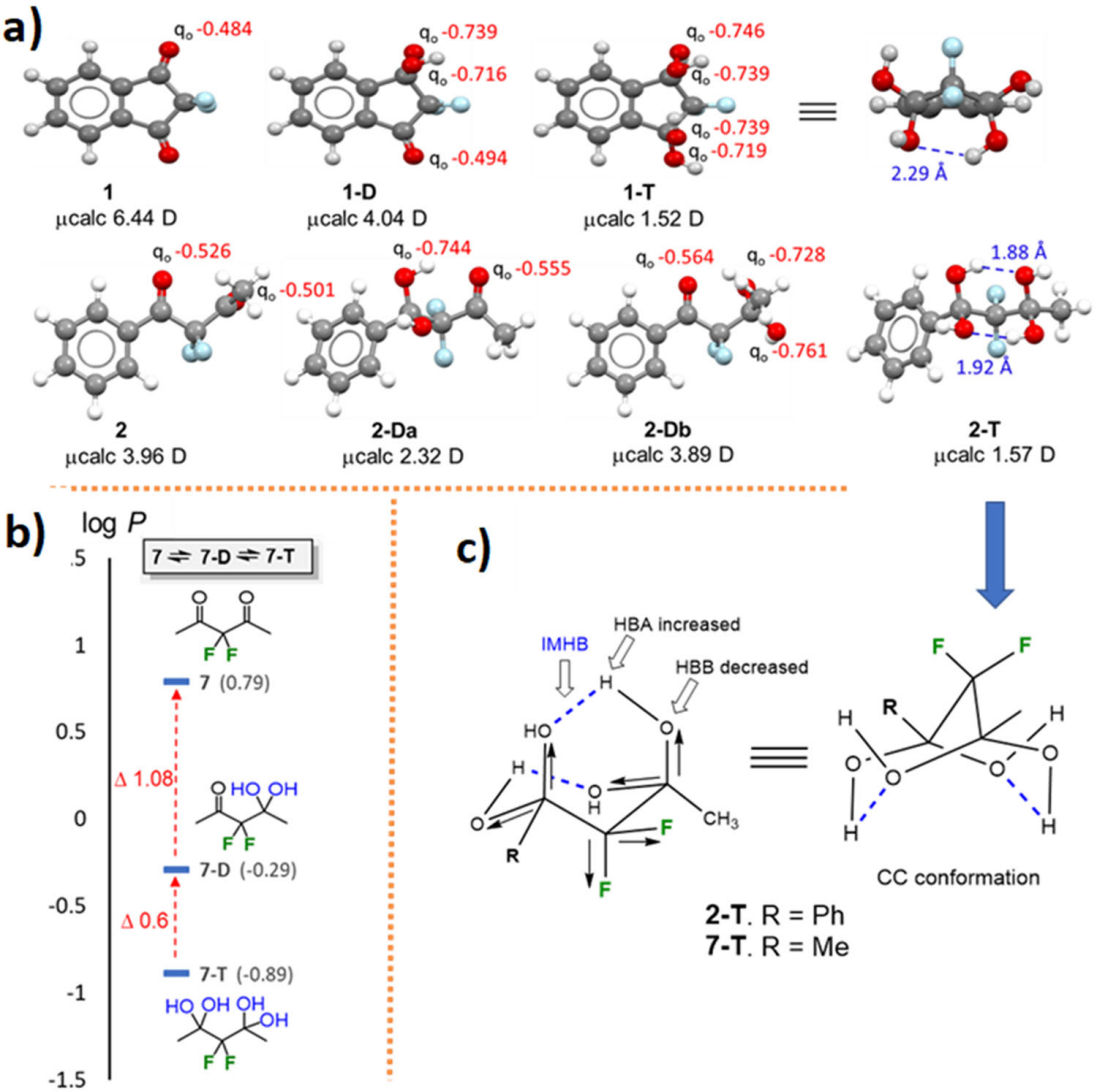
DFT results for FDKs 1 and 2. **a** Conformations, NBO charge distribution and overall polarity calculated for FDKs **1** and **2** and their hydrated forms using a DFT study. **b** Log *P* values of **7,** **7-D** and **7**-**T**. **c** The bicyclononane-like structures of **2-T** and **7-T**.

To study the origin of the above-mentioned phenomena, i.e. both equilibrium compositions and the differences in log *P* values of the various species measured, we employed DFT calculations (m062x/6-311 + +g(d,p) level of theory, gas phase). This included conformational analyses of FDKs **1** and **2** and their hydrated forms, their calculated overall polarity and NBO charge distribution of the oxygen functions which indicate their H-bonding capacity and possible intramolecular H-bonds. In addition, the keto-enol equilibria of FDKs **5** and **6** were also studied, both in terms of the relative conformational stability and the overall polarity (For the XYZ coordinates of the calculated species, see Supplemetary Data 1). Starting with the simplest rigid FDK **1**, we found that its diketo form adopts a planar conformation, and hence a relatively high dipole moment was calculated for this species (Fig. 10a, top row). The first hydration resulted in lowering of the polarity but increasing of the H-bond accepting capacity, as the partial negative charge on the oxygen atoms of the two hydroxyl groups is, as expected, higher than that of the oxygen atoms of the carbonyl function (ca. −0.74 versus ca. −0.49). Thus, the decrease in log *P* value from **1** to its gem-diol form **1-D** can be explained by the increase in the H-bond accepting capacity upon the first hydration of **1**. Further hydration of **1-D** leads to **1-T**, which adopts an envelope conformation of the fused five-membered ring, via single intramolecular H-bonding, and enjoys a further decrease in polarity. However, this decrease in polarity is significantly offset by the increase in H-bond accepting capacity of four hydroxyl groups (Fig. 10a, top row).

Turning to the asymmetric more complex FDK **2**, it was found that the diketo form adopts a conformation in which the two fluorine atoms are in an anti-position to the benzylic carbonyl (Fig. 10a, bottom row). Hydration at this less reactive position, as indicated by the above-mentioned results (**2-Da** regioisomer), led to a decrease in the overall polarity (increasing lipophilicity), and an increase of the H-bond accepting capacity (decreasing lipophilicity). This is, again, due to the replacement of one carbonyl (q_o_ −0.527) by two hydroxyl groups (q_o_ −0.744 and −0.758). The calculations of the other regioisomer **2-Db** reveals only a very slight decrease in the polarity upon this hydration, but again, significant increase in the H-bond accepting capacity resulting from a replacement of one carbonyl (q_o_ −0.501) by two hydroxyl groups (q_o_ −0.728 and −0.761). Thus, the considerable reduction in the log *P* values upon the first hydration of FDK **2** at one of the two carbonyls, can be explained by the significant increase in the H-bond accepting capacity of the gem-diols formed. The fact that the further hydration of these gem-diol forms, i.e. to tetrol **2-T**, led, surprisingly, to a much smaller decrease in lipophilicity compared to the transformation **1-D** → **1-T** (Δlog *P*_**2Da-2T**_ 0.27; Δlog *P*_**2Db-2T**_ 0.64 versus Δlog *P*_**1D-1T**_ 2.28, respectively) is quite interesting. A DFT study of tetrol **2-T** shows not only a decrease in the overall polarity (1.57 D) but also a preferred bicyclo[3.3.1]nonane-like CC-conformation dictated by two strong intramolecular H-bonds. Here, both H-bond acidity^40^ (increased) and basicity^31^ (decreased) are influenced by the β-fluorine atoms (Fig. 10c). Such intramolecular bonds, which are strongly supported by the above-mentioned ^19^F-DOSY-NMR results, can significantly decrease the interactions with the solvent, thus increasing the lipophilicity, leading to an overall lesser decrease in lipophilicity relative to **1-T**. This interesting effect was repeated when both equilibria and species specific log *P* values were studied for the symmetric analog of **2**, FDK **7** that was prepared for this purpose, and its hydrated forms **7**-**D** and **7**-**T** (Δlog *P*_**7-7D**_ 1.08; Δlog *P*_**7D-7T**_ 0.60, Fig. 10b; for details, see Figs. S17-S19, Supplementary Data 2).

**Fig. 11:**
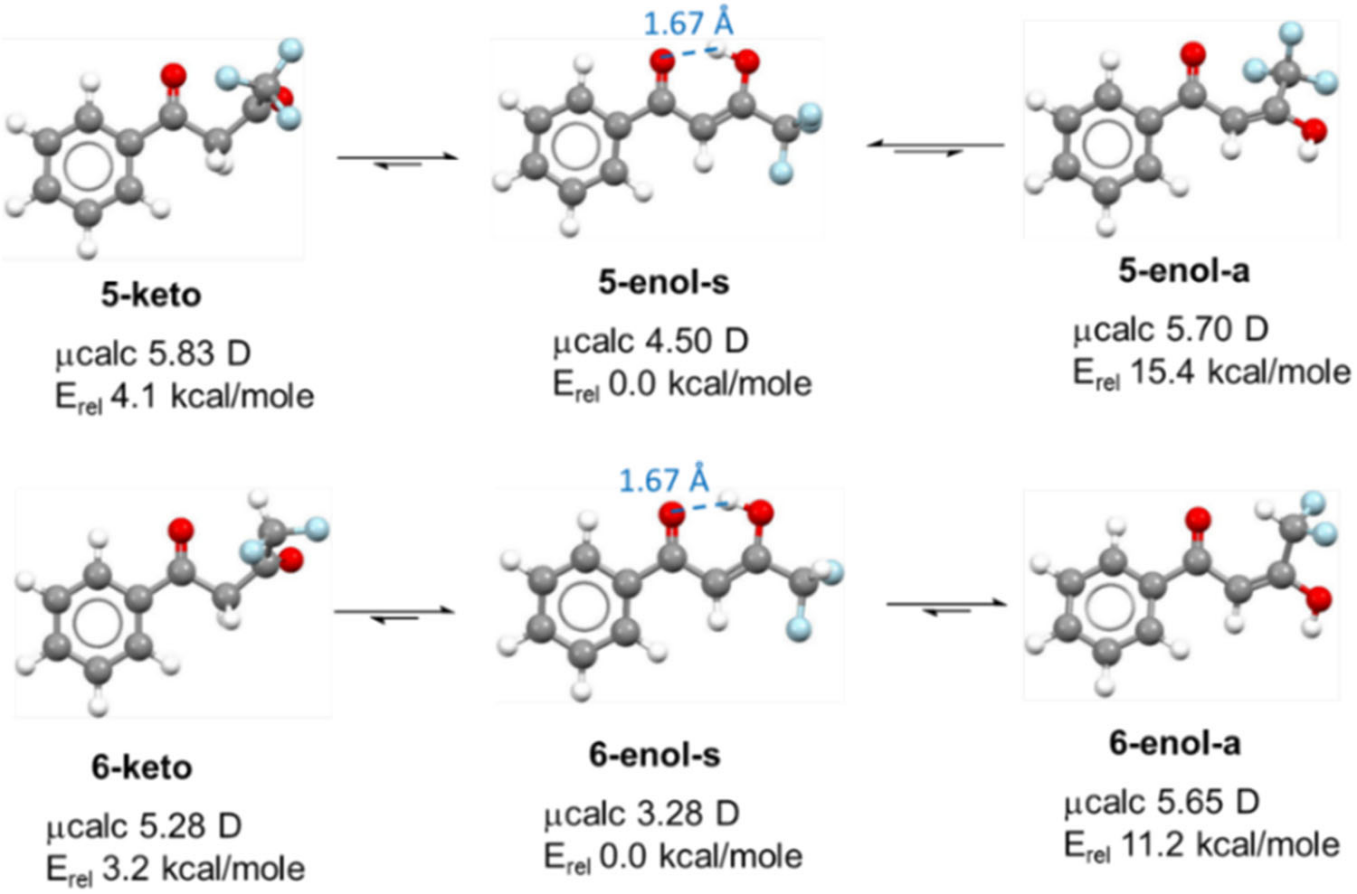
DFT results for FDKs 5 and 6. Conformations, NBO charge distribution and overall polarity calculated for FDKs **5** and **6** and their enol forms using a DFT study.

The conformations and keto-enol tautomerization as well as the NBO charge distributions and overall polarity of **5** and **6** were also calculated using the DFT study. As expected for both FDKs **5** and **6**, the enol forms **5-enol-s** and **6-enol**-**s**, in which a strong intramolecular H-bond exists, were found to be the stable tautomer relative to the keto form, by 4.1 and 3.2 kcal/mole, respectively (Fig. 11). Interestingly, the syn conformation that allows an intramolecular hydrogen bond (IMHB) between C = O^**…**^HO in **5-enol-s** is significantly more stable than the anti-conformation counterpart **5-enol-a** (15.4 Kcal/mole). However, since the CF_2_H group is a weak H-bond donor^25^, a smaller gap was found for the two enol forms of **6**, for which the anti-conformation **6-enol-a** can enjoy an alternative IMHB between the carbonyl and the difluoromethyl groups C = O^**…**^HCF_2_. Both stable enol forms **5-enol-s** and **6-enol-s** have the smallest overall dipole moments compared to the other species in equilibrium. These relative stabilities together with the IMHB that reduce the H-bond accepting capacity, and the lower polarity, clearly explain their high log *P* values.

### Water-membrane partitioning of 1 and its hydrated forms in equilibrium

**Fig. 12:**
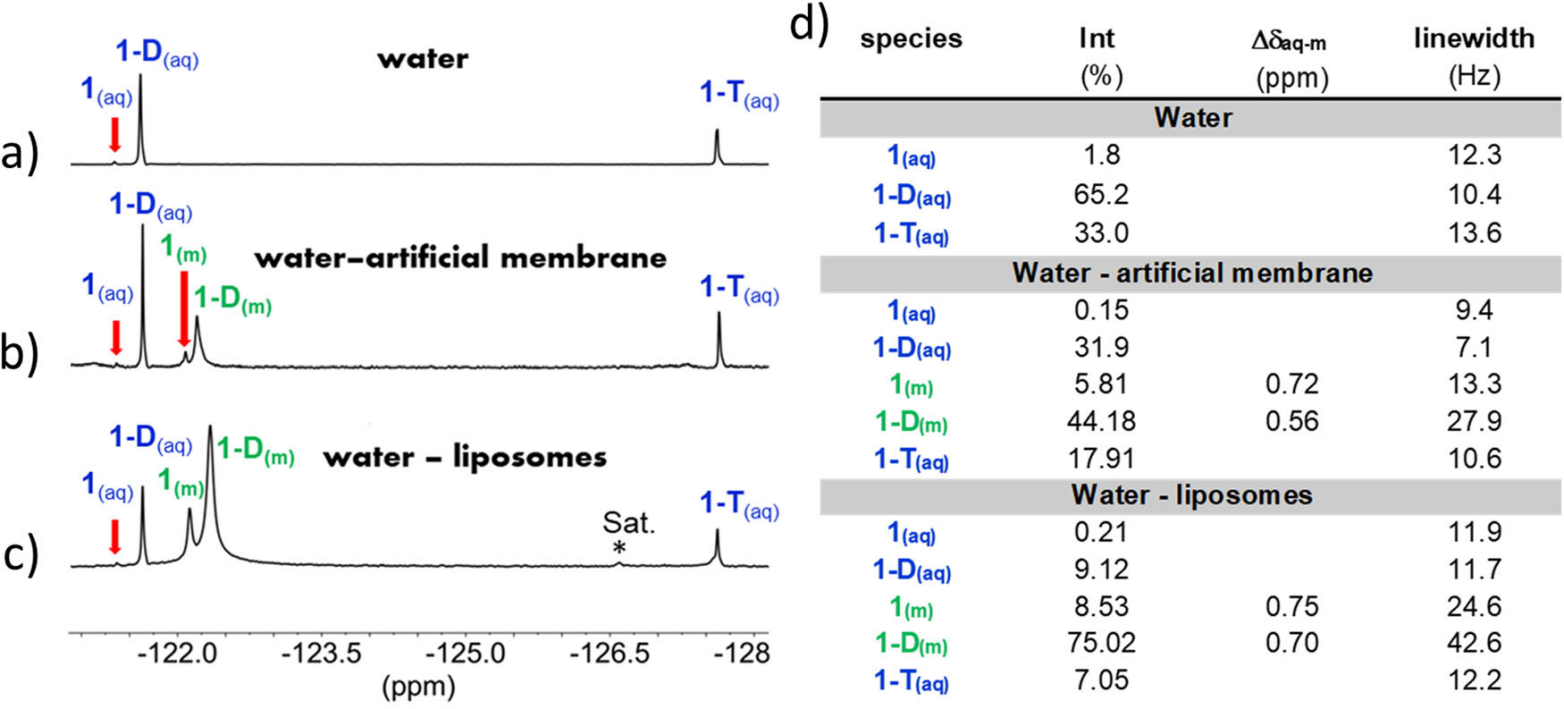
Membrane water partitioning of FDK 1 by solid-state ^19^F-MAS-NMR. **a** Spectrum in water. **b** Spectrum in a mixture of water with artificial membrane. **c** Spectrum in a mixture of water with liposomes. **d** Integrations and linewidths of the assigned signals.

The importance of species-specific log *P* values for drug design is born out of the better understanding we obtain as to the pharmacokinetic and pharmacodynamic behaviors expected from each species. As these species exist in an equilibrium, a range of log *P* values for “the molecule” is obtained, enabling adaptation of a drug containing such a warhead to changing environments. In the context of pharmacokinetics, body aqueous environments such as in the intestinal lumen and blood will lead to a higher ratio of the more hydrophilic species, while lipophilic or hydrophobic environments such as adipose tissue and protein hydrophobic binding pockets may accommodate more lipophilic species. In addition, the amphiphilic environment of the body’s membranes may be crossed by achieving a new equilibrium between the different possible species as needed. Such an alteration in the equilibrium of **1** and its hydrated forms **1D** and **1T**, for which the widest range of log *P* values was achieved, is well reflected in some preliminary results when an artificial membrane or liposomal suspensions were added to the aqueous solution containing these species. These heterogeneous mixtures were analyzed by solid-state ^19^F-MAS-NMR spectroscopy, and it can be clearly seen that the equilibrium composition in aqueous solutions of **1** and its hydrated forms changed in the presence of artificial membranes or liposomes (Fig. 12). Namely, the relatively more lipophilic species **1** and **1-D** accumulate within the membrane (**1**_**(m)**_ and **1-D**_**(m)**_) as indicated by the upfiled shift of their signals and the significant increase in their line width. On the other hand the concentration of the hydrophilic form **1-T**, which is mostly located in the aqueous phase, was reduced. It was very recently shown by Linclau’s group that lipophilicity modulations by fluorination correlate with membrane partitioning within families of fluorinated aliphatic compounds, using solid-state ^19^F MAS NMR and standard lipid vesicles^41^. Similarly, we show here for the first time that a correlation may also be valid in complex equilibrium systems, an issue that is currently under investigation in our laboratory.

### Reactions with nucleophilic amino acids Cys and Ser

**Fig. 13:**
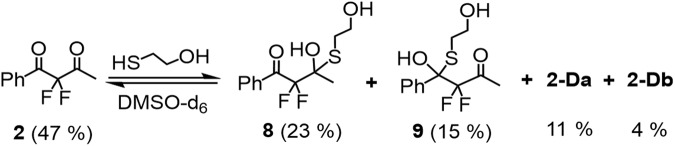
The reactivity of FDK 2 toward thiol. Reaction of **2** with 2-mercaptoethanol in DMSO-d_6_.

**Fig. 14:**
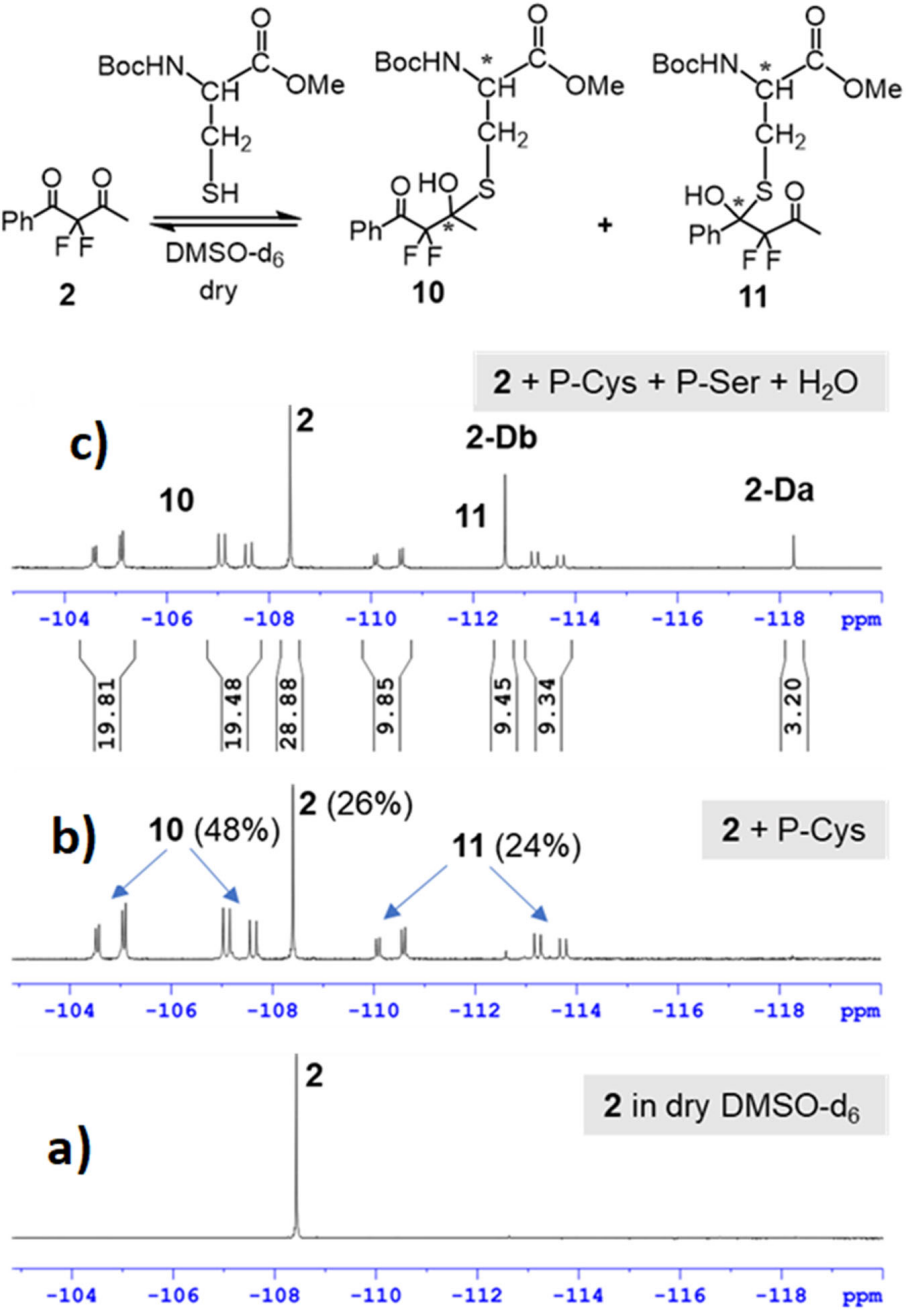
Reactions of FDK 2 with P-Cys in dry DMSO-d_6_. **a** ^19^F-NMR spectrum of **2** in dry DMSO-d_6_, **b** spectrum after the addition of 10 equivalents of P-Cys, and **c** spectrum after subsequent addition of P-Ser and water, 10 equivalent each.

Finally, the potential of the CF_2_(C = O)_2_ function to act as a multifaceted electrophilic warhead for targeted reversible covalent inhibitors, was chemically studied by a series of model reactions of **2** with selected nucleophiles, such as 2-mercaptoethanol and protected cysteine and serine in dilute solutions. Using ^19^F-NMR spectroscopy we focused on both the chemo- and regioselectivity in the reactions of this asymmetric FDK. We found that with DMSO-d_6_ as a solvent, the reaction of **2** with 2-mercaptoethanol (1 equivalent) occurred exclusively on the S-nucleophilic center forming both regioisomers of the corresponding hemi-thioketals **8** and **9** along with gem-diols **2-Da** and **2-Db** from residual water in this deuterated solvent (Fig. 13). Moreover, even in neat 2-mercaptoethanol as a solvent, the hemi-thioketal regioisomers **8** and **9** were observed almost exclusively, in 51 and 41%, respectively, together with ca. 4% of the corresponding hemiketal analogues (reaction with the OH group) and 4% of the FDK **2** (see Fig. S20, Supplementary Data 2). This chemoselectivity clearly indicates the superiority of nucleophiles bearing an SH group (such as the cysteine residue) over nucleophiles bearing the OH function (such as a serine amino acid) for FDK. Indeed, we found that the reaction of **2** with protected cysteine (P-Cys), the favored non-catalytic residue for targeted covalent inhibitors, led to a mixture containing the appropriate hemi-thioketal regioisomers **10** and **11** in 48 and 24%, respectively, together with 26% of FDK **2** (Fig. 14). Since a second asymmetric carbon is generated in the formation of these hemi-thioketals, two diastereoisomers are formed for each regioisomer with no selectivity, which is represented by two ABq systems in the ^19^F-NMR spectrum, owing to the diastereotopic nature of the fluorine atoms. Expectedly, no change was observed in the ^19^F-NMR spectrum of this reaction, after the addition of 10 equivalents of a protected serine analogue (P-Ser), as was also observed with a naïve solution of **2** with P-Ser. However, when 10 equivalents of water were added to the same mixture, the corresponding gem-diols **2-Da** and **2-Db** appeared together with hemi-thioketals **10** and **11**, previously observed, as shown in Fig. 14.

In this experiment the ratio between **10** and **11**, i.e. 2:1 respectively, did not change after the subsequent addition of water and immediate formation of **2-Da** and **2-Db**, indicating the reversibility of the process. Thus, in addition to the above-mentioned physicochemical features, the fact that the CF_2_(C = O)_2_ warhead in asymmetric FDKs such as **2** reacted predominantly with a protected cysteine at both electrophilic carbonyls to form the corresponding regioisomers **10** and **11** in equilibrium, provides additional value to this warhead. Hence, from a pharmacodynamic point of view, the two electrophilic centers to be attacked lend the warhead flexibility to react at an extended area holding a non-catalytic cysteine residue, following the molecular recognition of the parent drug in the binding site. This may, in some cases, alleviate the need for extensive linker search. Together, both features, i.e. the flexibility in the dual electrophilic centers to be attacked (pharmacodynamic effects) and the lipophilicities range that enables adaptation to the environment according to the degree of its lipophilicity/hydrophilicity (pharmacokinetics effect), grants this group (CF_2_(C = O)_2_) its potential to serve as a multifaceted warhead for reversible covalent drugs.

## Conclusions

In this report we described a thorough study of key physicochemical, molecular and chemical properties of a series of fluorinated geminal diketones and the multicomponent equilibrium systems they form in the presence of protic nucleophiles. We can conclude the following:

- The equilibria compositions of the FDKs **1-6** in water and octanol media, even for highly complex systems containing multiple species (up to 9 species) were studied using ^19^F- and ^13^C-NMR spectroscopy. When compounds containing the CF_2_(CO)_2_ function, in which both carbonyls are active (such as **1** and **2**), were dissolved in octanol saturated with water, all possible hydrated and hemiketal forms were detected and identified.
- Having identified the different species involved in the equilibrium in water and octanol saturated with water, a simultaneous and direct species-specific log *P* determination of these components was successfully achieved using the ^19^F-NMR-based method.
- A range of lipophilicities was found for each system, in some cases such as in FDKs **1** and **2**, spanning from lipophilic to hydrophilic species, indicating the possibility of adaptation to the environment, which may improve pharmacokinetic performance of drugs holding the CF_2_(CO)_2_ function.
- In addition to substantiating the signal assignment of the complex equilibrium system observed by FDKs **2** and **1** in both phases, ^19^F-DOSY-NMR spectroscopy enabled the exploration of inter and intramolecular hydrogen bonds, which strengthened our interpretation of the lipophilicity range observed.
- DFT studies revealed that the main factor which affects lipophilicity is the H-bond capacity of each species. Intramolecular H-bonds were clearly observed for both tetrols **1-T** and **2-T**. However, interestingly, the latter, more flexible one, may adopt a bicyclo[3.3.1]nonane-like CC-conformation dictated by two strong intramolecular H-bonds, which lead to a modest decrease in lipophilicity compared to the related gem-diols **2-Da** and **2-Db**. DFT studies of the relative conformational stability and the overall polarity of FDKs **5** and **6** and their enol forms, revealed that both enol forms **5-enol** and **6-enol** enjoy a higher stability relative to their keto counterparts and lower polarity, effects known to increase lipophilicity.
- A preliminary study of **1** and its hydrated forms **1-D** and **1-T** (for which a lipophilic to hydrophilic switch was observed) partitioning between water and artificial membrane or liposomes, using solid-state ^19^F-MAS-NMR indicated the possibility for adaptation to different environments.
- For a representative compound, the asymmetric FDK **2**, we have shown that it reacts with protected cysteine at both activated carbonyls to form two hemi-thioketal regioisomers, indicating the potential adaptability of the CF_2_(CO)_2_ warhead to noncatalytic cysteine residues in an active site environment which may affect pharmacodynamics.

The results described above are the basis for our conclusion that the CF_2_(CO)_2_ group has the potential to act as a novel type warhead for the design of reversible covalent drugs with a special ability to adapt to different environments and binding requirements.

## Methods

### General

Commercially available high-grade reagents and solvents were used without further purification. NMR spectra were recorded on a 11.7 T Bruker (AVANCE III HD) spectrometer (^1^H-NMR: 500 MHz; ^13^C-NMR: 125 MHz, ^19^F-NMR: 470.7 MHz) or a 7.0 T Bruker (AVANCE III HD) spectrometer (^1^H-NMR: 300 MHz; ^13^C-NMR: 75 MHz, ^19^F-NMR: 282 MHz). Chemical shifts are reported in parts per million (δ, ppm). ^1^H-NMR chemical shifts were referenced to the residual CDCl_3_ (δ = 7.26 ppm) or MeOD (δ = 3.31 ppm). In ^13^C-NMR measurements, the signal of CDCl_3_ (δ = 77.0 ppm) was used as reference. ^19^F-NMR chemical shifts were referenced to CCl_3_F as 0 ppm. High-resolution mass spectra were obtained with an LC-HRMS mass spectrometer operated in the positive electrospray ionization (ESI) mode. For information on the materials and the log *P* determination protocol, see Supplementary Methods.

### Diffusion measurements

^19^F-diffusion-NMR measurements were performed on an 11.7 T Bruker NMR spectrometer, operating at 470.7 MHz for ^19^F, equipped with a z-gradient system capable of producing a maximal pulsed gradient of about 50 G/cm. ^19^F-diffusion-NMR experiments were performed using the eddy currents delays (LED) pulse sequence. Sine-shaped pulse gradients, of 3 ms duration, were incremented from 1.0 to 49.0 G/cm in 16 steps, and the pulse gradient separation was 100 ms. The diffusion coefficients were extracted from: ln ${{{{{\rm{I}}}}}}/{{{{{{\rm{I}}}}}}}_{0}=-{{{{{{\rm{\gamma }}}}}}}^{2}{{{{{\rm{\delta }}}}}}^{2}{{{{{\rm{G}}}}}}^{2}(2/{{{{{\rm{\pi }}}}}})^{2}(\Delta {-}{{{{{\rm{\delta }}}}}}/4){{{{{\rm{D}}}}}}={-}{{{{{\rm{bD}}}}}}$, where I and I_0_ are the echo intensity, in the presence and absence of the gradient pulse, respectively, γ is the gyromagnetic ratio, G is the pulse gradient strength, 2/π is a geometrical correction factor due to the sine shape of the pulse gradients used, δ is the duration of the pulse gradient, Δ is the time interval between the leading edges of the pulse gradient used, and D is the diffusion coefficient. The diffusion coefficients were extracted from the slope of the plot of ln(I/I_0_) against the b-values. The diffusion NMR data was acquired at 299 °K. The given values represent means ± the standard deviation of the means, calculated by Bruker Dynamic Center software (2.3.3).

### Solid-State NMR measurements

^19^F-MAS-NMR measurements were carried out on an 11.7 T (500 MHz) Bruker spectrometer (Avance III HD) equipped with a 4 mm standard cross polarization (CP) magic-angle spinning (MAS) probe with zirconia rotors. Samples were spun at 10 kHz. Artificial membranes (commercial) were composed of cellulose membrane and Lecithine (S-100). Water-liposomes mixtures were prepared using aqueous solutions of multi-lamellar lipid vesicles composed of 1-palmitoyl-2-oleoyl-glycero-3-phosphocholine (POPC) as in Linclau’s recent publication^41^.

### Supplementary information

Supplementary Information

Description of Additional Supplementary Files

Supplemenrary data 1

Supplementary data 2

## Data Availability

The authors declare that the data supporting the study are available within the article and Supplementary Information. For experimental details and methods, see Supplementary Information. For XYZ coordinates of the DFT calculations, see Supplementary Data 1. For NMR spectra, see Supplementary Data 2.
