# Supplementary material for: Species-specific lipophilicities of fluorinated diketones in complex equilibria systems and their potential as multifaceted reversible covalent warheads
Source: Commun Chem. 2023 Sep 15;6:197. doi: 10.1038/s42004-023-01004-2 (PMC10504258; doi:10.1038/s42004-023-01004-2)
Supplement: Supplementary file 1 — Supplementary Information [file 42004_2023_1004_MOESM1_ESM.pdf]

Supplementary Information

Species-Specific Lipophilicities of Fluorinated Diketones in Complex Equilibria Systems and their Potential as Multifaceted Reversible Covalent Warheads

Ishay Columbus,[$] Lee Ghindes-Azaria,[$] Ido Michael Herzog, Eliav Blum, Galit Parvari, Yoav Eichen, Yoram Cohen, Eytan Gershonov, Eyal Drug, Sigal Saphier,* Shlomi Elias, Boris Smolkin and Yossi Zafrani*

[$] These authors contributed equally

Table of contents

1. **Supplementary Methods** **2**

Supplementary Method 1: Materials **2**

Supplementary Method 2: Detailed log *P* determination protocol **4**

2. **Supplementary Notes** **6**

Supplementary Note 1: Distribution of the DFK compounds in water and octanol **6**

Supplementary Note 2: Log *P* values - calculated vs. experimental **7**

Supplementary Note 3: Diffusion results of FDK 1 **8**

3. **Supplementary References** **9**

1. Supplementary Methods

Supplementary Method 1: Materials

Compounds **5** and **6** were purchased from commercial suppliers. Compounds **1**, **2** and **7** were prepared according to the literature procedures.[1] The products were obtained in pure form as indicated from their ^{1}H-, ^{13}C- and ^{19}F-NMR spectra, which fitted their previously reported data.[2] Compound **3** is a new compound, and its synthesis will be described in detail. Compound **4** was synthesized based on a modified literature procedure, as will be described.

2,2-difluoro-N-methyl-3-oxo-3-phenylpropanamide **(3):** Ethyl benzoylacetate **12** (150 µl, 0.86 mmol) was dissolved in acetonitrile (3 ml) and SelectFluor (644 mg, 1.8 mmol) was dissolved in water (3 ml) and added to the mixture. After stirring overnight, a mixture of mono and difluorinated compounds (3:1 respectively) was obtained according to ^{1}H- and ^{19}F-NMR spectra, and another portion of SelectFluor was added (322 mg, 0.86 mmol). Additional stirring for 48 h at room temperature and 16 h at 45 °C was required to obtain a mixture of mono and difluorinated products (1:9 respectively). Then, the reaction mixture was extracted with ethyl acetate, concentrated, dissolved in chloroform and filtered. Purification on silica using a gradient of 30% ethyl acetate in hexane provided pure ethyl benzoyldifluoroacetate **13** (74 mg, 38%), as indicated from their ^{1}H- and ^{19}F-NMR spectra, which fitted their previously reported data in the literature.[2] Next, **13** (74 mg, 0.33 mmol) was dissolved in dry ethanol (3.5 ml) under argon. Methyl amine (33 wt. % in absolute ethanol, 48 µl, 0.39 mmol) was added, and the reaction mixture was stirred for 16 h at room temperature and concentrated. Purification on silica using a gradient of 30% ethyl acetate in hexane provided the desired product **3** (45 mg, 65%) as indicated from the ^{1}H-, ^{13}C- and ^{19}F-NMR spectra.

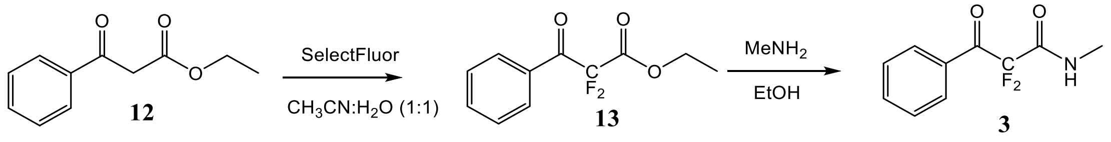

^{1}H-NMR (500MHz, $CDCl_3$): δ 8.19 (d, J = 8.0 Hz, 2H), 7.70 (t, J = 7.5 Hz, 1H), 7.56 (t, J = 8 Hz, 2H), 6.56 (s, 1H), 2.99 (d, J = 4.5 Hz, 3H); ^{13}C-NMR (125 MHz, $CDCl_3$): δ 187.6 (t, J =27.5 Hz), 162.2 (t, J = 27.5 Hz), 135.0, 131.8, 130.6 (t, J = 2.5 Hz), 128.9,

111.1 (t, J = 263.7 Hz), 26.9; ^{19}F-NMR (470.7 MHz): δ -108.2. HRMS (ESI+) m/z calculated for $C_{10}H_9F_2NO_2$ [M+H+] 214.06741, found 214.06714.

Benzoylfluoroacetone (**4**): Compound **4** was prepared according to a modified procedure described by Tang *et al*. Benzoylacetone **14** (250 mg, 1.54 mmol) was dissolved in acetonitrile (3 ml), and SelectFluor (492 mg, 1.38 mmol) was added as solid in portions. After stirring for 2 h, chloroform was added and the mixture was filtered. Purification of this mixture on silica using a gradient of 10% ethyl acetate in hexane provided a mixture of the keto and enol forms of **4** (60 mg), as indicated from their ^{1}H- and ^{19}F-NMR spectra, which fitted their previously reported data in the literature.[3]

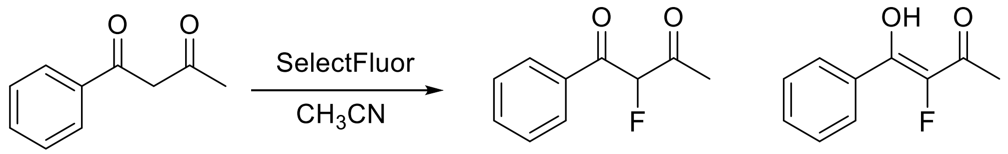

14 **4-keto** **4-enol**

Supplementary Method 2: Materials Detailed log *P* determination protocol

Mixture preparation and partitioning. To a 10 mL pear-shaped flask was added octanol (HPLC grade, ca. 2 mL), the measured compound (ca. 10-30 mg), trifluoroethanol as the reference compound (ca. 10-30 mg), and water (double distilled or the relevant buffer, ca. 2 mL). The resulting biphasic mixture was stirred at room temperature for 2 h and then left to stand for 2 h to enable complete phase separation.

Sample preparation. Using two 1 mL disposable syringes, aliquots of ca. 0.5–0.7 mL were carefully sampled from each layer. For sampling the water aliquot, ca. 0.05 mL volume of air was pumped to the syringe before inserting the needle into the solution. While moving through the upper octanol layer, the air was gently pushed out, as a very small amount of octanol may otherwise penetrate into the needle causing a large measurement error for lipophilic compounds. Upon reaching the water phase, all the air bubbles were pushed slowly out of the syringe, the aliquot was sampled, and the needle was quickly removed from the solution. Then a small volume of the water phase was discarded to ensure the removal of any trace of octanol from the needle, leaving ca. 0.5-0.6 mL volume of sample in the syringe. The needle was carefully wiped with a dry tissue, and the sample was injected into an NMR tube. The addition of a deuterated solvent (for lock and shim purposes) was not necessary, since good results were obtained without deuterated solvents. For every compound, duplicates of each phase prepared and measured.

NMR measurement. ^{19}F {^{1}H} (fluorine with proton decoupling) NMR was initially run with standard parameter conditions for chemical shift identification. The frequency offset point (O1P) is centered between the diagnostic F signals. From our experience in these compounds, in most cases the t1 values span between 2-5 s. Hence, delay time (D1) was set to 30 s (5×t1) for most of the samples. In highly diluted samples, D1 of 5-15 s was chosen in order to acquire more scans per time unit. The spectral width (SW) was 300 ppm. The number of transients (NS) was at least 32 (but could be increased significantly if higher SNR was required). A calibrated shim file for the probe was loaded for each experiment.

Data processing and log *P* calculation. Data were processed using Topspin NMR software. The obtained FID file was reprocessed using the following conditions: LB was set to 2-10, Zero Filling from 64k to 256k points and then Fourier transform followed by manual phasing. The integration ratio was obtained by manual integration (bias correction was applied via adjusting tilt and slope if integral curve was not parallel to the baseline). The log *P* was calculated via the equation: $\log Px_{(o/w)} = \log P_{(ref)} + \log (Ix_{(o)}/Ix_{(w)} * Iref_{(aq)}/Iref_{(o)})$, where Ix is the average integral of each phase (taken relatively to the internal reference) calculated from the duplicate.

2. Supplementary Notes

Supplementary Note 1: Materials Distribution (%) of the DFK compounds in water and octanol

diketone	species	In water	In octanol saturated with water
**1**	**1**	1.8	7.1
	**1-D**	65.2	17.6
	**1-T**	33.0	0.06
	**1-HK**		74.5
	**1-HKD**		0.42
	**1-DHK**		0.26
**2**	**2**	2.1	22.7
	**2-Da**	30.1	4.8
	**2-Db**	62.2	24.5
	**2-T**	5.6	0.57
	**2-HKa**		20.9
	**2-HKb**		21.2
	**2-HKaD**		2.9
	**2-HKbD**		2.0
	**2-DHK**		0.38
**3**	**3**	6.8	36.6
	**3-D**	93.2	12.3
	**3-HK**		51.1
**4**	**4-keto**		48.3
	**4-enol**		51.7
	**4-Da**	19.1	
	**4-Db**	80.9	
**5**	**5-enol**	6.7	49.0
	**5-D**	93.3	32.8
	**5-HK**		18.2
**6**	**6-enol**	2.8	73.2
	**6-D**	97.2	15.4
	**6-HK**		11.4
**7**	**7**	0.6	2.2
	**7-D**	87.8	34.1
	**7-T**	11.6	1.5
	**7-HK**		56.3
	**7-HKD**		4.2
	**7-DHK**		1.8

Table S1. Distribution (%) of the DFK compounds in water and in octanol saturated with water (integration from the ^{19}F-NMR spectra).

Supplementary Note 2: Log *P* values - calculated vs. experimental

Compound	Structure	Clog *P*	Milog *P*	Experimental
**1**	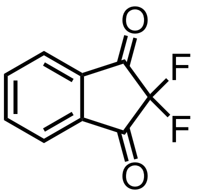	2.64	1.44	1.73
**1-D**	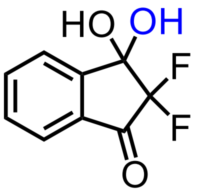	1.26	0.67	0.44
**1-T**	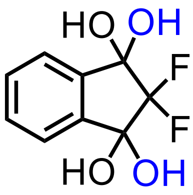	-0.25	-0.11	-1.84
**2**	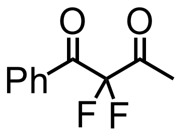	2.53	1.65	2.62
**2-Da**	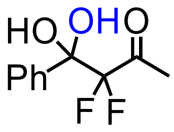	1.22	0.87	0.96
**2-Db**	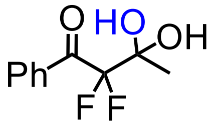	1.5	1.26	1.33
**2-T**	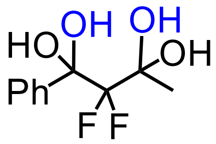	-0.4	0.48	0.69

Table S2. Log *P* values of DFKs **1** and **2**: predictions by Clog *P* and Milog *P* vs. MNR experimental results.

Supplementary Note 3: Materials Diffusion results of FDK 1

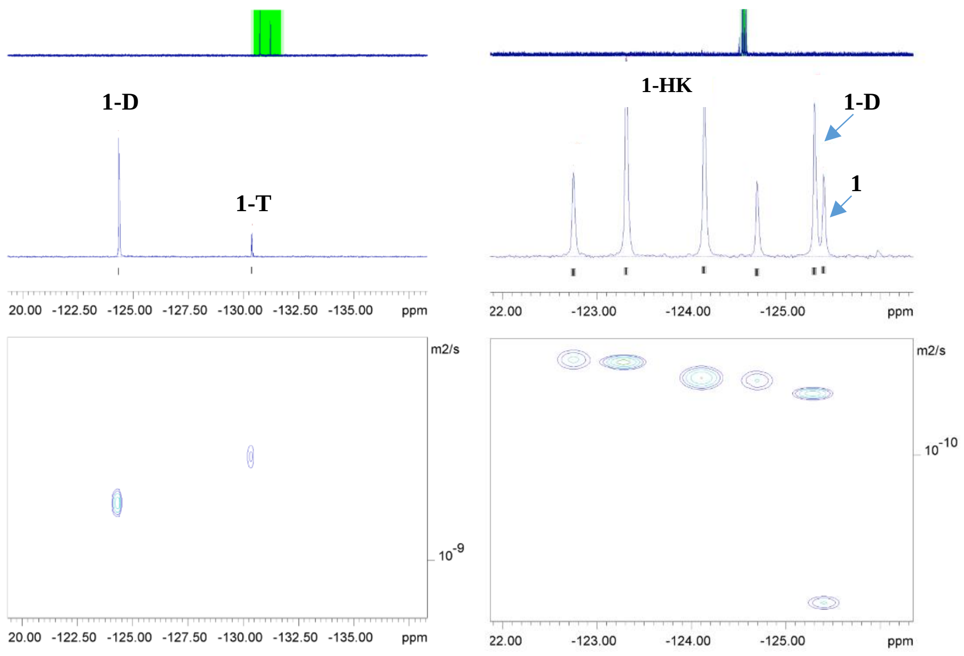

DOSY spectra (^{19}F-NMR) of the equilibria mixtures obtained in water (left) and octanol (right) in the log *P* determination experiment of diketone **1**.

	D in water x 10^{-10} (m^2/s)	D in octanol x 10^{-10} (m^2/s)
**1**	nd	1.83 ± 0.05
**1-D**	6.52 ± 0.56	0.77 ± 0.02
**1-T**	4.52 ± 0.55	nd
**1-HK**	nd	0.71 ± 0.04
**1-HKD**	nd	nd
**1-DHK**	nd	nd

Table S3. Diffusion coefficients of the species involved in log *P* determination of FDK **1**.

3. Supplementary references

1. Tang, L., Yang, Z., Jiao, J., Cui, Y., Zou, G., Zhou, Q., Zhou, Y., Rao, W. & Ma, X. Chemoselective mono- and difluorination of 1,3-dicarbonyl compounds. *J. Org. Chem*. **84**, 10449−10458 (2019).
2. Sloop, J. C., Boyle, P. D., Fountain, A. W., Gomez, C., Jackson, J. L., Pearman, W. F., Schmidt, R. D. & Weyand, J. Novel fluorinated indanone, tetralone and naphthone derivatives: Synthesis and unique structural features. *Appl. Sci*. **2**, 61-99 (2012).
3. Zeng, X., Lu, Z., Liu, S., Hammond, G. B. & Xu, B. Gold-catalyzed fluorination of alkynyl esters and ketones: Efficient access to Fluorinated 1,3-dicarbonyl compounds. *Adv. Synth. Catal*. **359**, 4062–4066 (2017).
