# Supplementary material for: Species-specific lipophilicities of fluorinated diketones in complex equilibria systems and their potential as multifaceted reversible covalent warheads
Source: Commun Chem. 2023 Sep 15;6:197. doi: 10.1038/s42004-023-01004-2 (PMC10504258; doi:10.1038/s42004-023-01004-2)
Supplement: Supplementary file 2 — Description of Additional Supplementary Files [file 42004_2023_1004_MOESM2_ESM.pdf]

Description of Additional Supplementary

Files

File name: Supplementary data 1

Description: DFT studies and XYZ coordinates for the calculated species

File name: Supplementary data 2

Description: NMR spectra of the FDKs **1-7** and their hydrated and ketal forms and explanations of the assignments of the different species
