# Supplementary material for: Species-specific lipophilicities of fluorinated diketones in complex equilibria systems and their potential as multifaceted reversible covalent warheads
Source: Commun Chem. 2023 Sep 15;6:197. doi: 10.1038/s42004-023-01004-2 (PMC10504258; doi:10.1038/s42004-023-01004-2)
Supplement: Supplementary file 3 — Supplemenrary data 1 [file 42004_2023_1004_MOESM3_ESM.pdf]

DFT studies and XYZ coordinates for the calculated species

All calculations were performed using the Gaussian 09 Revision D.01 program,[1] at the #m062x/6-311++g(d,p) level of theory, for single gas-phase species, with accompanying frequencies analyses for verification of minima points on the potential energy surface. These frequency analyses were used to derive relative energies of species, which are reported in comparison to each other based on the differences of sums of electronic and thermal free energies. NBO analyses[2] were also performed using the Gaussian program, following the optimization of each species. All graphics of the simulated species were created using the Mercury 3.9 program, build RC1 of the Cambridge Crystallographic Data Center.

XYZ geometry coordinates of the species (from the calculation output) follow below:

Compound **1**

Center Number	Atomic Number	Atomic Type	Coordinates (Angstroms) X	Y	Z
1	6	0	0.000022	1.872962	1.418360
2	6	0	0.000101	0.683071	0.698059
3	6	0	0.000101	0.683071	-0.698059
4	6	0	0.000022	1.872962	-1.418360
5	6	0	-0.000093	3.061422	-0.701374
6	6	0	-0.000093	3.061422	0.701374
7	1	0	0.000033	1.856627	2.501553
8	1	0	0.000033	1.856627	-2.501553
9	1	0	-0.000190	4.007395	-1.229571
10	1	0	-0.000190	4.007395	1.229571

11	6	0	-0.000104	-1.646674	0.000000
12	9	0	1.091525	-2.435154	0.000000
13	9	0	-1.092397	-2.434291	0.000000
14	6	0	0.000177	-0.702240	-1.229128
15	6	0	0.000177	-0.702240	1.229128
16	8	0	0.000394	-1.062849	-2.369018
17	8	0	0.000394	-1.062849	2.369018

Compound **1-D**

Center Number	Atomic Number	Atomic Type	Coordinates (Angstroms) X	Y	Z
1	6	0	0.203805	1.934054	1.386507
2	6	0	0.056156	0.733712	0.708416
3	6	0	-0.112498	0.708574	-0.672886
4	6	0	-0.147263	1.881596	-1.422264
5	6	0	0.000252	3.084582	-0.747831
6	6	0	0.175506	3.108232	0.641534
7	1	0	0.331251	1.946215	2.462244
8	1	0	-0.278950	1.839837	-2.496984
9	1	0	-0.017251	4.018040	-1.297190
10	1	0	0.285603	4.062339	1.143774
11	6	0	0.183976	-1.558978	0.066954

12	9	0	1.512245	-1.858900	-0.059811
13	9	0	-0.483260	-2.717943	0.149233
14	6	0	-0.219569	-0.686523	-1.147991
15	6	0	-0.014543	-0.654458	1.315857
16	8	0	-0.520456	-1.112766	-2.224052
17	8	0	-1.286354	-0.821684	1.836039
18	1	0	-1.350859	-1.717653	2.190278
19	8	0	0.916962	-0.914449	2.321011
20	1	0	1.784558	-1.054693	1.925197

Compound **1-T**

Center Number	Atomic Number	Atomic Type	Coordinates (Angstroms) X	Y	Z
1	6	0	2.002921	1.426688	-0.007090
2	6	0	0.825575	0.695942	-0.060188
3	6	0	0.836032	-0.692776	-0.033998
4	6	0	2.026115	-1.398988	0.050550
5	6	0	3.211959	-0.672916	0.102153
6	6	0	3.201224	0.724217	0.072779
7	1	0	1.980037	2.509956	-0.020935
8	1	0	2.019572	-2.481538	0.086385

9	1	0	4.158414	-1.195702	0.173741
10	1	0	4.138943	1.265148	0.118245
11	6	0	-1.391714	-0.013434	0.340727
12	9	0	-1.446237	0.010502	1.697228
13	9	0	-2.665712	-0.007159	-0.107370
14	6	0	-0.568997	-1.243050	-0.141825
15	6	0	-0.589839	1.219550	-0.150933
16	8	0	-0.933226	-1.564227	-1.459675
17	8	0	-0.896624	1.459532	-1.492078
18	1	0	-1.789246	1.828525	-1.522235
19	8	0	-0.870388	2.375652	0.572324
20	1	0	-0.856966	2.166122	1.513451
21	1	0	-0.876684	-0.770021	-2.007887
22	8	0	-0.742821	-2.372093	0.629089
23	1	0	-1.541719	-2.814888	0.320176

Compound **2**

Center Number	Atomic Number	Atomic Type	Coordinates (Angstroms) X	Y	Z
1	6	0	-3.015221	-1.279720	0.155057
2	6	0	-1.650477	-1.021400	0.112929
3	6	0	-1.199985	0.294254	-0.024607

4	6	0	-2.120558	1.342737	-0.120890
5	6	0	-3.480176	1.078020	-0.082457
6	6	0	-3.927308	-0.234626	0.056531
7	1	0	-3.366194	-2.298515	0.263009
8	1	0	-0.948984	-1.841423	0.188937
9	1	0	-1.747650	2.354346	-0.227146
10	1	0	-4.192891	1.889826	-0.161131
11	1	0	-4.990620	-0.441962	0.087043
12	6	0	0.241516	0.650741	-0.065058
13	6	0	1.295964	-0.481642	-0.041432
14	6	0	2.737272	0.072606	-0.166627
15	6	0	3.342684	0.618848	1.093562
16	1	0	2.679170	1.373320	1.521411
17	1	0	3.455679	-0.183100	1.826386
18	1	0	4.311090	1.055668	0.860797
19	8	0	0.646356	1.785531	-0.111001
20	8	0	3.275517	0.031850	-1.233496
21	9	0	1.180381	-1.165656	1.137110
22	9	0	1.051080	-1.355022	-1.037706

Compound **2-Da**

Center Number	Atomic Number	Atomic Type	Coordinates (Angstroms) X	Y	Z
1	6	0	3.319883	0.948737	0.508304
2	6	0	1.983927	1.164469	0.190441
3	6	0	1.215791	0.121739	-0.322808
4	6	0	1.784387	-1.138304	-0.503688
5	6	0	3.121341	-1.348856	-0.186812
6	6	0	3.890543	-0.305915	0.318856
7	1	0	3.916469	1.762483	0.902874
8	1	0	1.530566	2.137551	0.327925
9	1	0	1.176273	-1.948978	-0.886561
10	1	0	3.561029	-2.328671	-0.331137
11	1	0	4.932562	-0.471683	0.566083
12	6	0	-0.248928	0.345371	-0.645015
13	6	0	-1.149056	-0.059332	0.548808
14	6	0	-2.645113	0.004182	0.161997
15	6	0	-3.424518	-1.270953	0.197937
16	1	0	-2.972677	-1.969834	-0.511253
17	1	0	-3.355301	-1.726998	1.187728
18	1	0	-4.459316	-1.067995	-0.067424
19	8	0	-0.435313	1.685843	-0.951455
20	8	0	-3.089390	1.072845	-0.171388

21	9	0	-0.837239	-1.311370	0.962046
22	9	0	-0.928307	0.777160	1.586361
23	1	0	-1.381782	1.889213	-0.919093
24	8	0	-0.716798	-0.500283	-1.670183
25	1	0	-0.215437	-0.301265	-2.468722

Compound **2-Db**

Center Number	Atomic Number	Atomic Type	Coordinates (Angstroms) X	Y	Z
1	6	0	-3.243181	-1.356114	0.040083
2	6	0	-1.887090	-1.054751	0.014618
3	6	0	-1.475054	0.281112	-0.010461
4	6	0	-2.428880	1.304682	-0.010176
5	6	0	-3.779718	0.997431	0.017869
6	6	0	-4.187010	-0.335005	0.042475
7	1	0	-3.562283	-2.390978	0.057530
8	1	0	-1.162921	-1.857548	0.011383
9	1	0	-2.089047	2.333039	-0.033224
10	1	0	-4.515855	1.791946	0.018666
11	1	0	-5.243507	-0.576196	0.062840
12	6	0	-0.046303	0.686223	-0.055487

13	6	0	1.043221	-0.414736	-0.005108
14	6	0	2.514359	0.078040	0.004775
15	6	0	2.877296	0.742922	1.318716
16	1	0	2.256086	1.620991	1.495533
17	1	0	2.758711	0.037268	2.140290
18	1	0	3.922314	1.044300	1.250778
19	8	0	3.334061	-1.031764	-0.146035
20	9	0	0.820530	-1.184459	1.093333
21	9	0	0.864659	-1.220924	-1.091403
22	1	0	3.215258	-1.366201	-1.042738
23	8	0	2.712634	0.905603	-1.111101
24	8	0	0.298229	1.843280	-0.133120
25	1	0	2.169327	1.696051	-1.000197

Compound **2-T**

Center Number	Atomic Number	Atomic Type	Coordinates (Angstroms) X	Y	Z
1	6	0	0.016881	-0.277996	0.731565
2	6	0	-0.935839	0.047365	-0.456609
3	6	0	-2.454582	0.124348	-0.142626
4	1	0	-3.652493	-1.031030	0.862944
5	6	0	1.466531	-0.103459	0.313596

6	6	0	2.057305	1.158403	0.350673
7	6	0	2.200455	-1.201239	-0.129546
8	6	0	3.382174	1.317486	-0.038737
9	1	0	1.476498	2.012699	0.677235
10	6	0	3.524606	-1.037436	-0.520325
11	1	0	1.730245	-2.175440	-0.156741
12	6	0	4.117556	0.219887	-0.474010
13	1	0	3.838063	2.299798	-0.005513
14	1	0	4.094314	-1.894511	-0.859395
15	1	0	5.150119	0.344983	-0.778098
16	8	0	-0.350449	0.654464	1.733938
17	1	0	0.223006	0.507504	2.494351
18	9	0	-0.580329	1.236729	-0.998881
19	9	0	-0.734663	-0.899920	-1.405814
20	6	0	-3.262797	0.209447	-1.423125
21	1	0	-4.314405	0.324808	-1.153023
22	1	0	-2.955508	1.082328	-1.998535
23	1	0	-3.129600	-0.693564	-2.015392
24	8	0	-2.763470	1.238590	0.622891
25	1	0	-2.124375	1.296550	1.347117
26	8	0	-2.736961	-1.073906	0.567874
27	8	0	-0.157319	-1.581194	1.166471
28	1	0	-1.109077	-1.749880	1.23277

Compound **5-keto**

Center Number	Atomic Number	Atomic Type	Coordinates (Angstroms) X	Y	Z
1	6	0	3.682959	-1.372994	-0.175819
2	6	0	2.331441	-1.180054	0.086905
3	6	0	1.812670	0.113289	0.166275
4	6	0	2.655639	1.211666	-0.019080
5	6	0	4.002358	1.016923	-0.285260
6	6	0	4.516575	-0.275912	-0.363093
7	1	0	4.083835	-2.377456	-0.234914
8	1	0	1.691587	-2.042700	0.232071
9	1	0	2.232759	2.206746	0.048614
10	1	0	4.654235	1.869352	-0.432628
11	1	0	5.569313	-0.428183	-0.570287
12	6	0	0.371366	0.381537	0.449334
13	6	0	-0.568320	-0.819783	0.590290
14	6	0	-1.980004	-0.329181	0.800257
15	6	0	-2.773010	-0.023258	-0.492550
16	8	0	-0.058637	1.500691	0.575247
17	8	0	-2.511912	-0.209044	1.859877
18	1	0	-0.504077	-1.449076	-0.302336
19	1	0	-0.278492	-1.410443	1.462209

20	9	0	-3.195125	-1.191718	-1.008987
21	9	0	-2.001322	0.558860	-1.414971
22	9	0	-3.825198	0.740101	-0.267962

Sum of electronic and thermal Free Energies= -835.071227 (Hartree/particle).

Compound **5-enol-s**

Center Number	Atomic Number	Atomic Type	Coordinates (Angstroms) X	Y	Z
1	6	0	-3.543493	-1.660421	-0.146691
2	6	0	-2.234652	-1.193935	-0.175521
3	6	0	-1.973523	0.169228	-0.021014
4	6	0	-3.035862	1.059011	0.160671
5	6	0	-4.340047	0.589930	0.199381
6	6	0	-4.595207	-0.770972	0.045241
7	1	0	-3.741390	-2.717482	-0.275632
8	1	0	-1.429152	-1.898626	-0.338562
9	1	0	-2.815739	2.113672	0.270826
10	1	0	-5.159164	1.282987	0.347805
11	1	0	-5.614553	-1.137679	0.072601
12	6	0	-0.594663	0.732992	-0.059224
13	6	0	0.565313	-0.152553	0.026243

14	6	0	1.801664	0.387340	-0.029672
15	6	0	3.054381	-0.463884	0.053499
16	8	0	-0.436249	1.952437	-0.151602
17	8	0	2.092840	1.662326	-0.160068
18	1	0	0.466603	-1.217454	0.153888
19	9	0	3.796837	-0.315951	-1.044182
20	9	0	2.759801	-1.760754	0.179403
21	9	0	3.798127	-0.108317	1.102070
22	1	0	1.224312	2.141265	-0.190662

Sum of electronic and thermal Free Energies= -835.077783 (Hartree/particle).

Compound **5-enol-a**

Center Number	Atomic Number	Atomic Type	Coordinates (Angstroms) X	Y	Z
1	6	0	3.555588	1.348187	-0.555653
2	6	0	2.210029	1.138832	-0.273269
3	6	0	1.776506	-0.105714	0.185180
4	6	0	2.698124	-1.141128	0.354481
5	6	0	4.041482	-0.927942	0.082495
6	6	0	4.470972	0.317600	-0.372003
7	1	0	3.888507	2.312121	-0.921089

8	1	0	1.496897	1.938758	-0.436740
9	1	0	2.337760	-2.103251	0.698711
10	1	0	4.756509	-1.729945	0.220823
11	1	0	5.520178	0.482832	-0.587385
12	6	0	0.335990	-0.389651	0.477780
13	6	0	-0.559978	0.783233	0.684338
14	6	0	-1.864404	0.811458	0.399124
15	6	0	-2.624861	-0.285294	-0.342297
16	8	0	-0.070520	-1.522826	0.588140
17	8	0	-2.702208	1.816862	0.705342
18	1	0	-0.125549	1.658304	1.158863
19	9	0	-3.704516	0.230025	-0.936316
20	1	0	-2.254630	2.459204	1.265285
21	9	0	-3.042582	-1.232252	0.496285
22	9	0	-1.883407	-0.849750	-1.291900

Sum of electronic and thermal Free Energies= -835.053400 (Hartree/particle).

Compound **6-keto**

Center Number	Atomic Number	Atomic Type	Coordinates (Angstroms) X	Y	Z
1	6	0	3.469439	-1.210085	-0.613060

2	6	0	2.102347	-1.121862	-0.377279
3	6	0	1.558706	0.046361	0.159551
4	6	0	2.394299	1.125657	0.458710
5	6	0	3.757512	1.035942	0.222055
6	6	0	4.295919	-0.132341	-0.313790
7	1	0	3.888061	-2.118218	-1.029297
8	1	0	1.468810	-1.968283	-0.615170
9	1	0	1.953328	2.023462	0.874674
10	1	0	4.403124	1.874205	0.454429
11	1	0	5.361451	-0.201733	-0.498609
12	6	0	0.098822	0.192699	0.431630
13	6	0	-0.818223	-0.983842	0.119404
14	6	0	-2.250995	-0.643442	0.466779
15	6	0	-2.885442	0.545125	-0.277437
16	1	0	-2.869564	1.451790	0.327158
17	8	0	-0.361414	1.209839	0.897502
18	8	0	-2.894529	-1.238151	1.281300
19	1	0	-0.745087	-1.221648	-0.946685
20	1	0	-0.525232	-1.862930	0.696788
21	9	0	-4.157046	0.239374	-0.607347
22	9	0	-2.200916	0.774469	-1.431886

Sum of electronic and thermal Free Energies= -735.809952 (Hartre/particle).

Compound **6-enol-s**

Center Number	Atomic Number	Atomic Type	Coordinates (Angstroms) X	Y	Z
1	6	0	-3.241403	-1.656932	-0.180428
2	6	0	-1.930400	-1.195941	-0.154411
3	6	0	-1.669798	0.168560	-0.011953
4	6	0	-2.735928	1.065143	0.100163
5	6	0	-4.043034	0.602243	0.082409
6	6	0	-4.297282	-0.760220	-0.057746
7	1	0	-3.437571	-2.715669	-0.298275
8	1	0	-1.121406	-1.906791	-0.263710
9	1	0	-2.515953	2.120770	0.200804
10	1	0	-4.865215	1.301393	0.175844
11	1	0	-5.318544	-1.122361	-0.073747
12	6	0	-0.286325	0.726730	0.012211
13	6	0	0.862771	-0.161063	0.146136
14	6	0	2.106648	0.369389	0.154822
15	6	0	3.355041	-0.469425	0.294419
16	8	0	-0.123882	1.948054	-0.068237
17	8	0	2.399570	1.650421	0.056286
18	1	0	0.757618	-1.227342	0.256984
19	9	0	4.081612	-0.378815	-0.845153

20	9	0	3.036552	-1.772377	0.476647
21	1	0	1.533188	2.127378	-0.026858
22	1	0	3.977155	-0.135362	1.127391

Sum of electronic and thermal Free Energies= -735.815138 (Hartree/particle).

Compound **6-enol-a**

Center Number	Atomic Number	Atomic Type	Coordinates (Angstroms) X	Y	Z
1	6	0	3.499818	1.357907	0.354287
2	6	0	2.133348	1.131991	0.231015
3	6	0	1.657965	-0.148548	-0.055556
4	6	0	2.561624	-1.201028	-0.215704
5	6	0	3.925537	-0.970778	-0.109938
6	6	0	4.395574	0.309138	0.176193
7	1	0	3.864218	2.349981	0.591937
8	1	0	1.440397	1.949965	0.390221
9	1	0	2.172099	-2.190666	-0.422508
10	1	0	4.624992	-1.786742	-0.246023
11	1	0	5.460809	0.487744	0.264447
12	6	0	0.195675	-0.458849	-0.160534
13	6	0	-0.711703	0.663444	-0.487810

14	6	0	-2.038529	0.652557	-0.283588
15	6	0	-2.809852	-0.460566	0.394044
16	8	0	-0.199958	-1.597799	-0.007870
17	8	0	-2.869120	1.641902	-0.637820
18	1	0	-0.273258	1.540944	-0.951925
19	9	0	-3.554029	-1.103892	-0.539985
20	1	0	-2.392433	2.327651	-1.116854
21	9	0	-3.671174	0.092303	1.284567
22	1	0	-2.174119	-1.179002	0.900523

Sum of electronic and thermal Free Energies= -735.797213 (Hartree/particle).

References

1. Frisch, M. J. *et al*. Gaussian 09, revision D.01; Gaussian, Inc.: Wallingford, CT, 2013.
2. Reed, A. E.; Weinhold, F. Natural Localized Molecular Orbitals. *J. Chem. Phys*. **1985**, *83*, 1736-1740.
