# Supplementary material for: Species-specific lipophilicities of fluorinated diketones in complex equilibria systems and their potential as multifaceted reversible covalent warheads
Source: Commun Chem. 2023 Sep 15;6:197. doi: 10.1038/s42004-023-01004-2 (PMC10504258; doi:10.1038/s42004-023-01004-2)
Supplement: Supplementary file 4 — Supplementary data 2 [file 42004_2023_1004_MOESM4_ESM.pdf]

NMR spectra of the FDKs and their hydrated and ketal forms

Explanation of the signal assignments for 1 and its hydrate and ketal forms. Starting from the simple ^{19}F-NMR spectrum of **1** in water (Figure S1, bottom), we observe three separate signals. The diketone **1** at -124.0 ppm (the most downfield) and two other signals of the hydrates forms, diol **1-D** (-124.3 ppm) and tetrol **1-T** (-130.3 ppm), with **1-T** expected to be most upfield. The ratio of **1-D** to **1-T** is ~2:1, while **1** appears as a very small signal (less than 2%), which can now be used for confirmation of the assignment, using the ^{13}C-NMR spectrum.

Since diketone **1** is almost fully hydrated and was observed as a very small signal in the ^{19}F-NMR spectrum, it was expected that at the ^{13}C-NMR spectrum it would not be observed. Looking at the ^{13}C-NMR spectrum (Figure S2, bottom), we observe only one carbonyl signal (triplet) assigned to the only carbonyl containing compound in meaningful amounts in the mixture, i.e. **1-D**. Nine signals of aromatic carbons were observed, six belong to the asymmetric **1-D** and three to the symmetric **1-T.** Additionally, two triplets of CF_2 are observed at 112.9 ppm and 122.7 ppm with a ratio of ~2:1. However, the gem-diol carbon ($C(OH)_2$) signals appear at a ratio of 1:1 at 92.7 ppm and 95.3 ppm. The fact that the ratio is 1:1 shows that our assumption that the ratio between the amounts of **1-D** and **1-T** was 2:1 is correct, since **1-T** holds two gem-diol carbons, while **1-D** only holds one such carbon, leading to a signal ratio of 1:1.

Once these assignments were established, we turned to the spectra in octanol. In the ^{19}F-NMR spectrum (Figure S1, top) three compounds are observed in large quantities, and three more represent small signals. The large signals show two singlets and one AB quartet. The two very close singlets are assigned to **1** (-125.2 ppm) and **1-D** (-125.3 ppm). The AB quartet (centered at -123.6 ppm) stems from the fluorine atoms residing next to a prochiral carbon center, formed by the hemi-ketal formation of the carbonyl with octanol. Three structures from the mixture, will have such prochiral centers, i.e **1-HK**, **1-HKD** and **1-DHK**. The AB quartet at -123.6 ppm was assigned to **1-HK** as will be explained below using the ^{13}C-NMR spectrum. The three small signals (Figure S1 top, inset) present a singlet assigned to **1-T** (based on prior assignment in water), an AB quartet centered at -127.7 ppm that was assigned to **1-HKD**, while the symmetry of **1-DHK** led to two singlets (-129.0 ppm, -129.5 ppm) of the two diastereomers formed in this case.

Turning to the ^{13}C-NMR spectrum, we observe three carbonyl triplet signals. Two of the signals correspond to the previously assigned (in water) **1** and **1-D.** The third triplet must belong to the only other carbonyl containing molecule in the mixture, i.e. **1-HK**. Since this signal intensity is larger than that of **1-D**, this leads to the conclusion that **1-HK** is a major component in the mixture, explaining the assignment of the large AB quartet signal in the ^{19}F-NMR spectrum to this species.

At this point, all signals in the ^{19}F-NMR spectrum were assigned to the different species, and therefore log *P* experiments could be conducted.

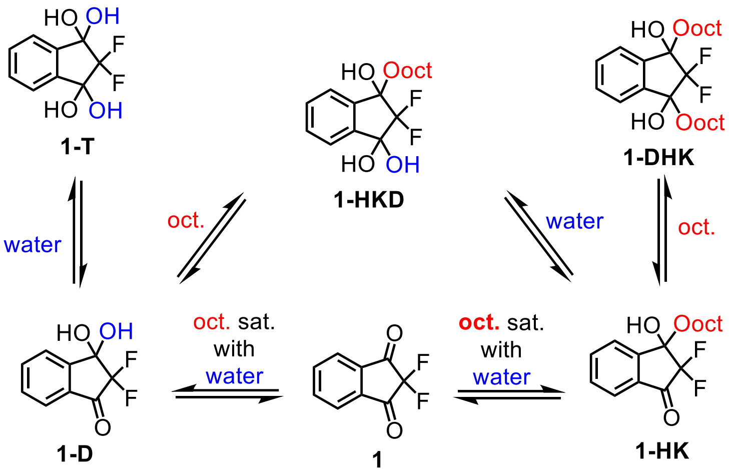

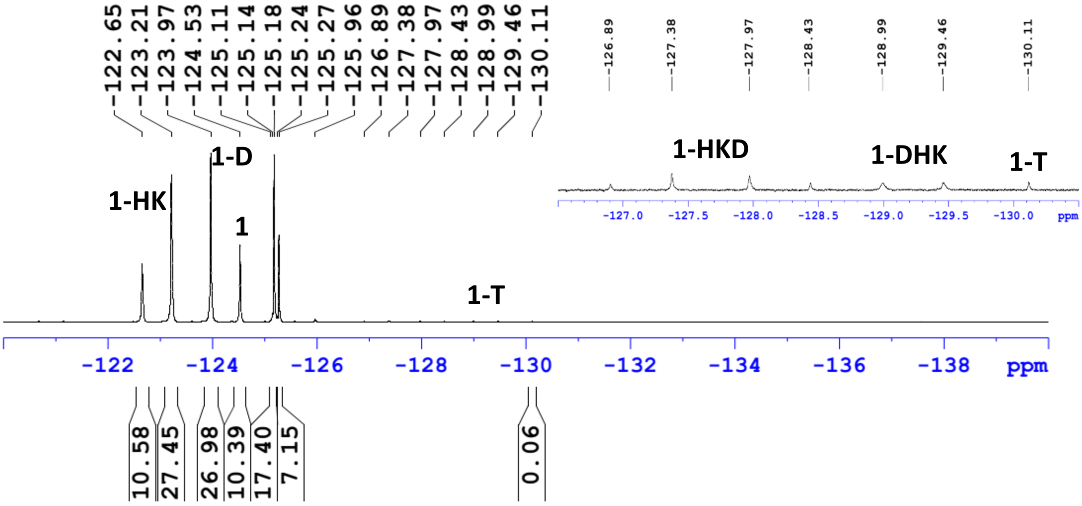

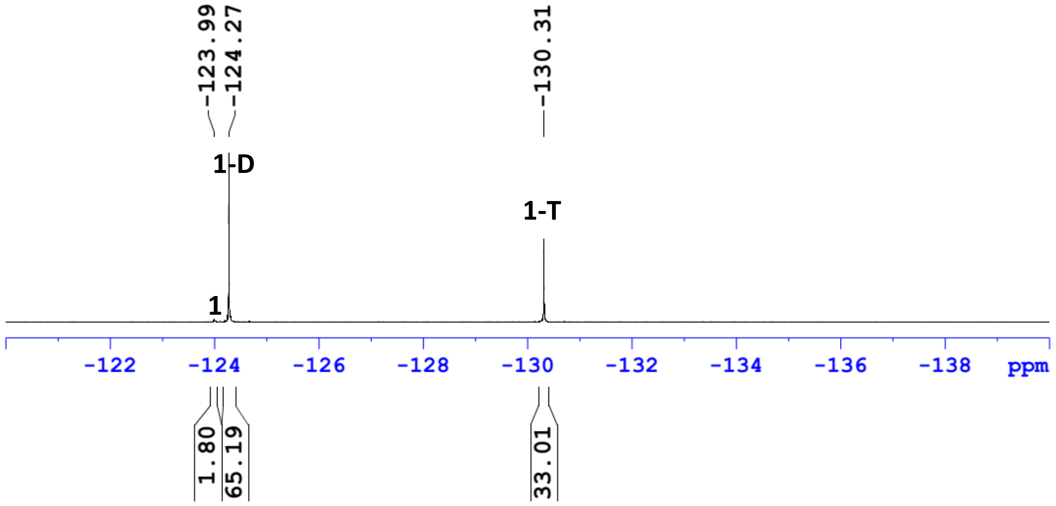

Figure S1. ^{19}F-NMR spectra of diketone **1** in octanol saturated with water (top) and in water (bottom).

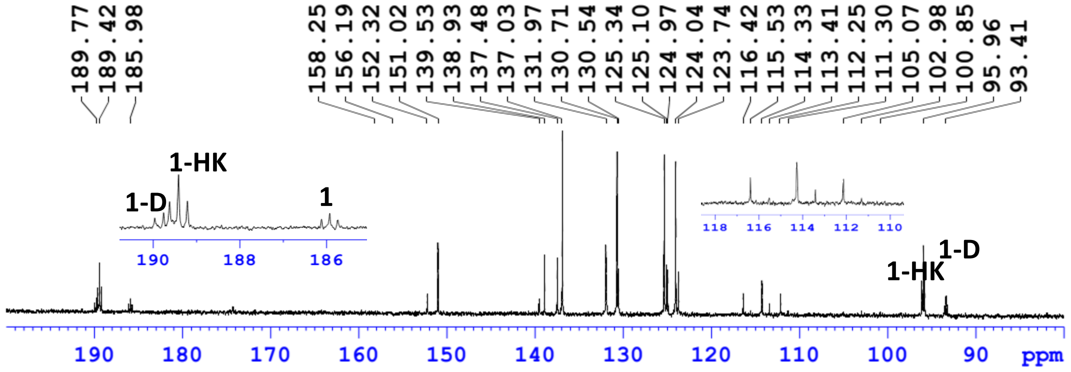

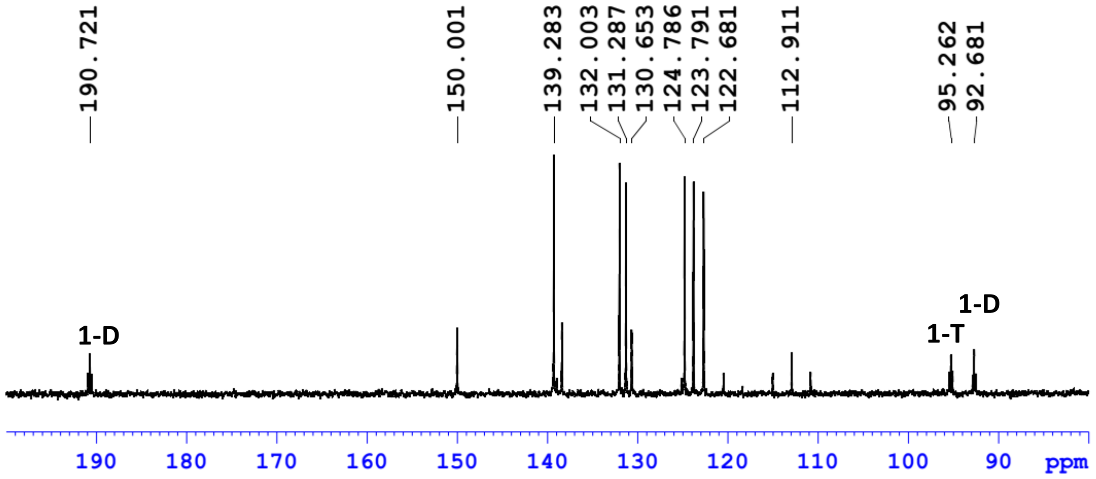

Figure S2. ^{13}C-NMR spectra of diketone **1** in octanol saturated with water (top) and in D_2O (bottom).

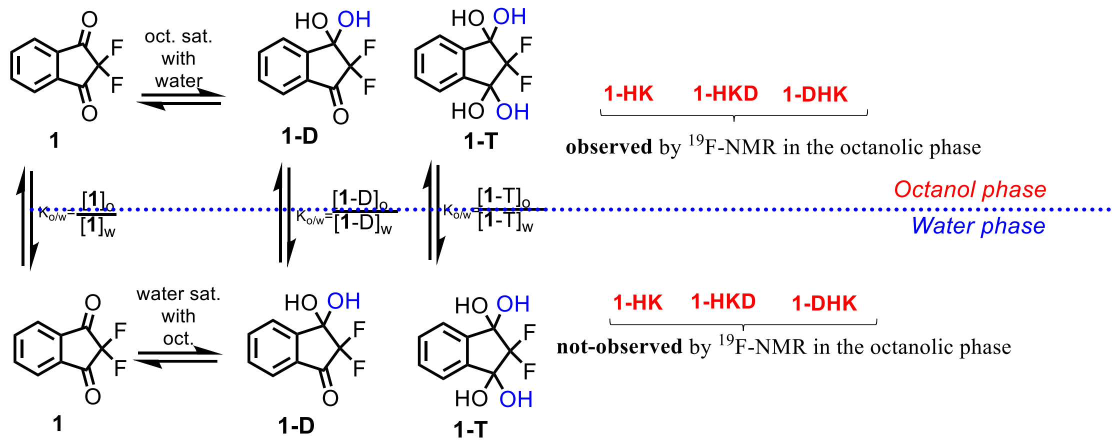

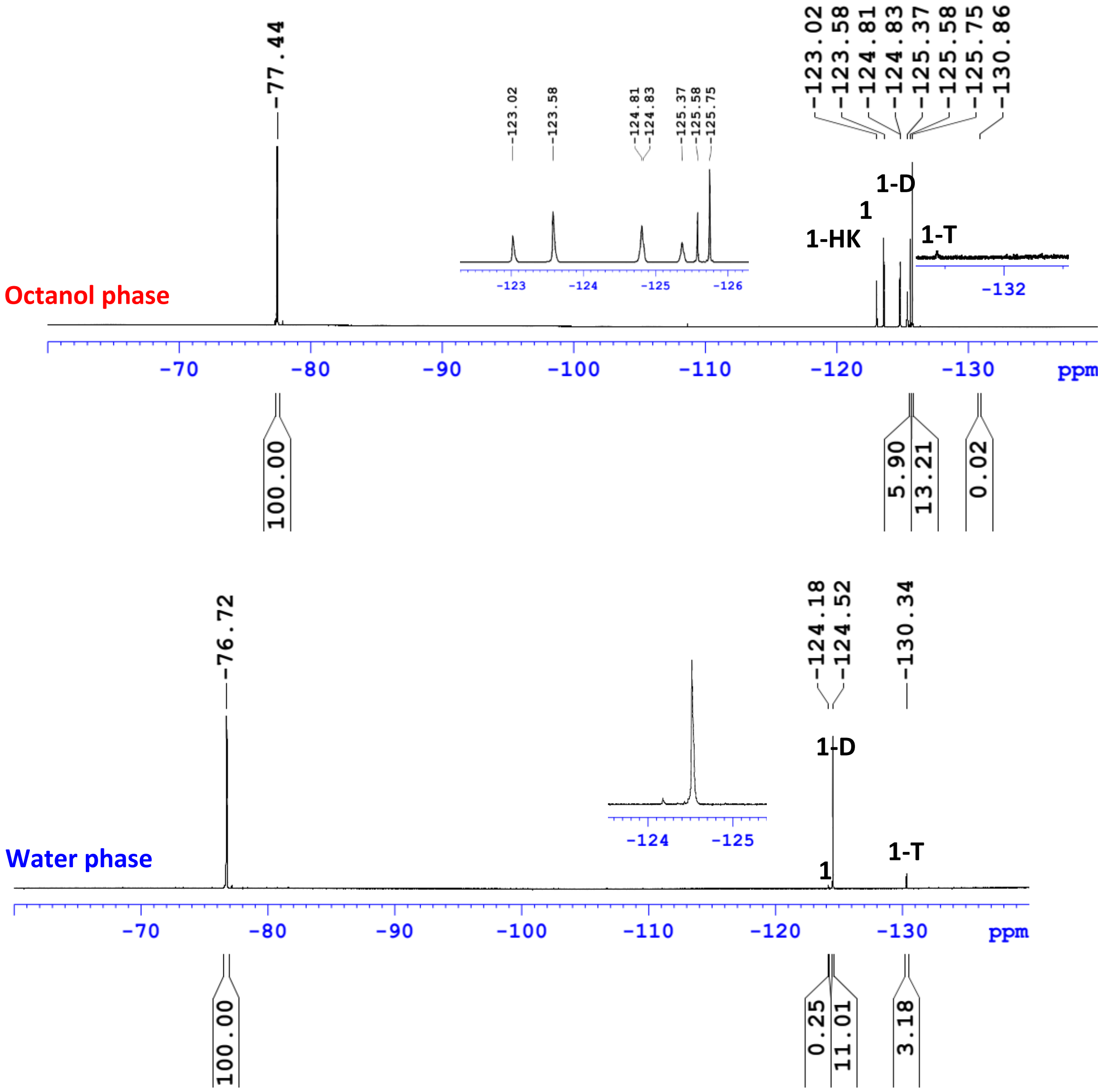

Figure S3. ^{19}F-NMR spectra of diketone **1** in the octanol phase (top) and in the water phase (bottom) after a stir-flask experiment.

Explanation of the signal assignments for 2 and its hydrate and ketal forms. Starting from the simple ^{19}F-NMR spectrum of **2** in water (Figure S4, bottom), we observe four separate signals. These are assigned to **2** (-109.0 ppm), **2-Db** (-114.1 ppm), **2-Da** (-120.2 ppm) and **2-T** (-122.0 ppm). The assignment of the weak diketone **2** signal is based upon the ^{19}F-NMR spectrum of this compound in $CDCl_3$. As explained above, a tetrol is expected to be more upfield than the diol, and therefore the **2-T** was assigned to the most upfield signal. The distinction between diol **2-Db** (more intense) and **2-Da** (weaker) was based on their ^{13}C-NMR signals. Looking at the ^{13}C-NMR spectrum (Figure S5, middle), we observe two carbonyl triplet signals belonging to the two keto-diols. The more downfield and weaker intensity signal was assigned to **2-Da** (202.3 ppm) with a carbonyl close to the terminal methyl, while the more upfield and intense signal (X1.4) was assigned to **2-Db** (192.8 ppm) with the carbonyl near the aromatic ring. This was based on the difference between two measured carbonyl chemical shifts of model compounds, trifluoroacetone (189.6 ppm) and trifluoroacetophenone (180.4 ppm). These show that a chemical shift difference of 9 ppm is expected, with the "near aromatic" carbonyl expected to be more upfield. Thus, more nucleophilic attack and gem-diol formation on the less hindered carbonyl occurred, which is also what would be expected since the carbonyl conjugated with the aromatic ring is also less prone to nucleophilic attack. Going back to the ^{19}F-NMR, the higher intensity signal, at -114.1 ppm belongs to **2-Db**.

Having assigned all the signals in the ^{19}F-NMR spectrum of the mixture in water, we now turn to the more complex NMR spectrum in octanol (Figure S4, top). This spectrum contains nine species, with five of the species more dominant and four minor components. First, we assign the compounds that were already assigned in the spectrum in water. These are: compound **2** (-109.0 ppm), **2-Db** (-113.3 ppm), **2-Da** (-119.0 ppm) and **2-T** which is a minor component at -121.3 ppm. The other two dominant species are two AB quartets which, since they are quartets, were assigned to the two hemi-ketals **2-Hkb** and **2-Hka**. The downfield AB quartet centered at -113.5 ppm was attributed to **2-HKb**, and the upfield AB quartet centered at -118.4 ppm was attributed to **2-HKa**, based on the chemical shifts of the equivalent gem-diols **2-Da** and **2-Db**.

We are left with the identification of three minor species; **2-HKaD**, **2-HKbD**, **2-DHK**, and all three species appear as AB quartets more upfield, as now there is no carbonyl moiety (Figure S4 top, inset). These hemiketal-diols are not present in the water phase, and therefore their log *P* values could not be determined. However, in order to complete the picture, we assigned the signals as follows: The more upfield and low intensity AB quartet signal (-122.6 ppm) is attributed to the double hemi-ketal **DHK**, which is likely to be the rarer component. As for **2-**

HKbD and **2-HKaD**, the chemical shift difference between them is much smaller than their hemiketal counterparts, yet we assume the same order, and therefore expect **2-HKbD** to be more downfield and **2-HKaD** more upfield, centered at -119.5 ppm and -120.8 ppm, respectively.

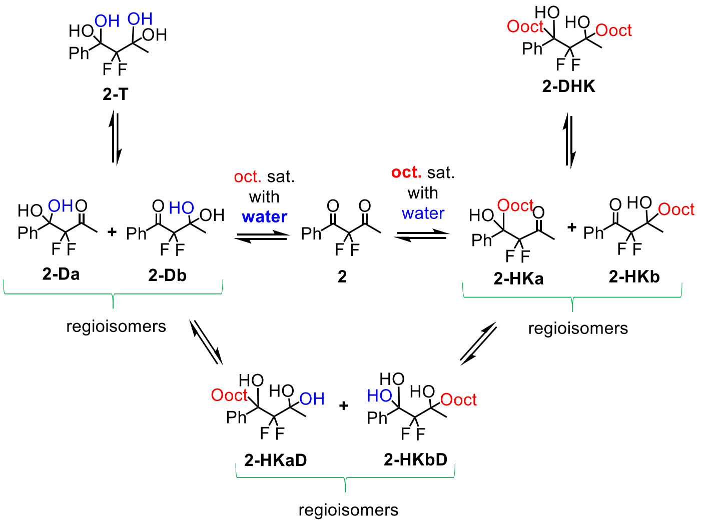

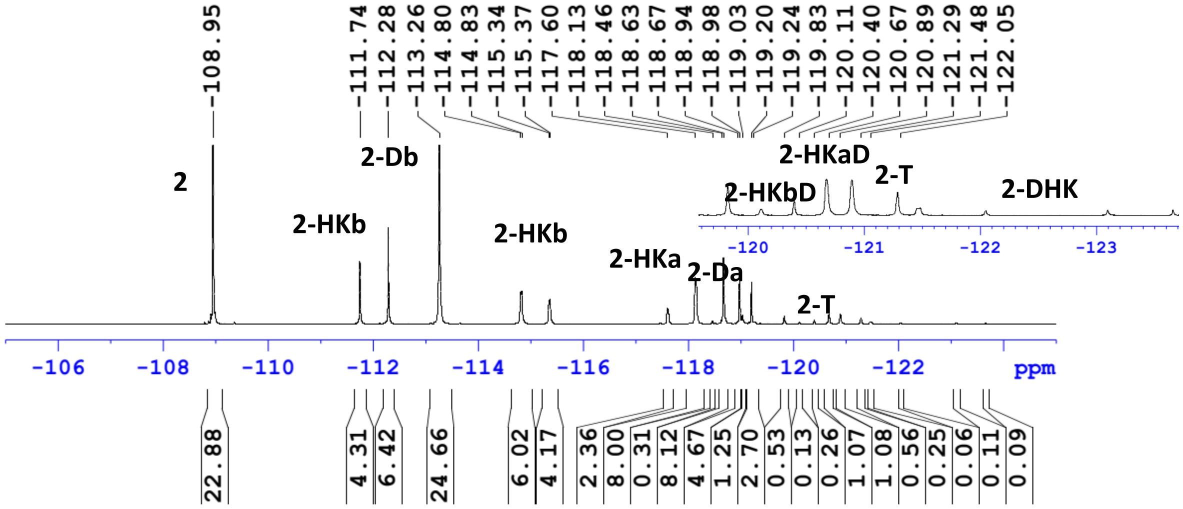

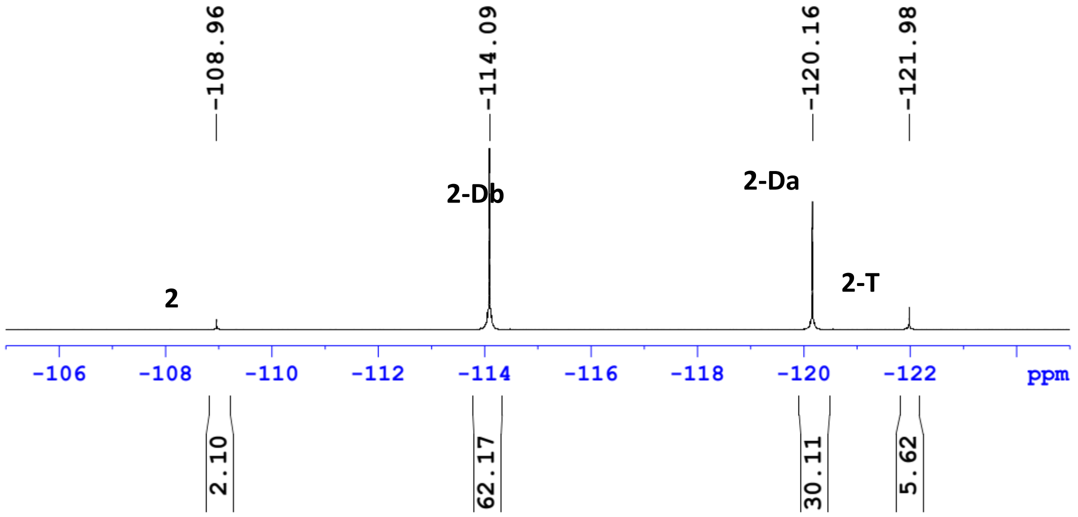

Figure S4. ^{19}F-NMR spectra of diketone **2** in octanol saturated with water (top) and in water (bottom).

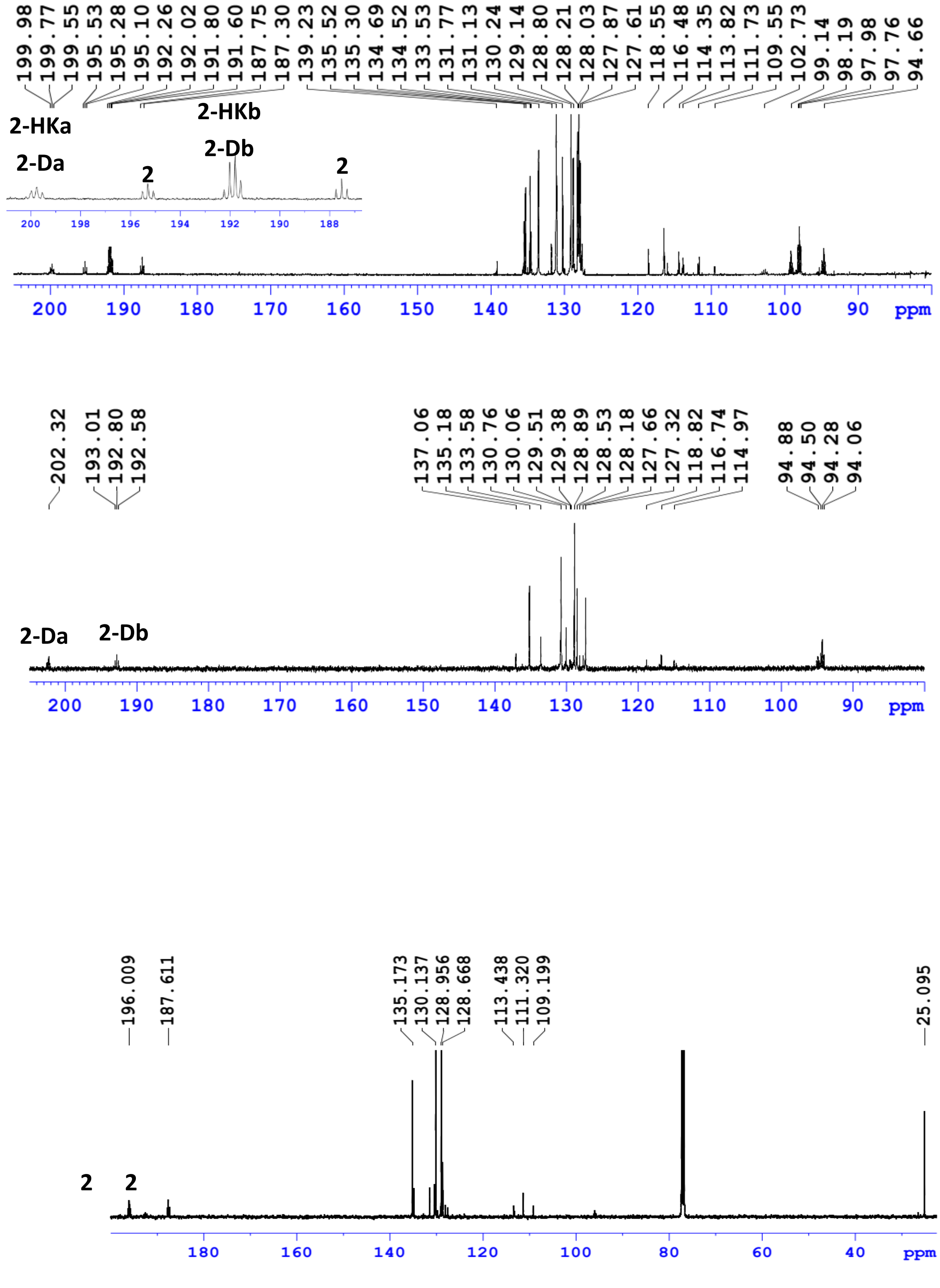

Figure S5. ^{13}C-NMR spectra of diketone **2** in octanol saturated with water (top), in water (middle) and in $CDCl_3$ (bottom).

Explanation of the signal assignments for 3 and its hydrate and ketal forms.

In the aqueous solution only the two species **3** and **3-D** were observed in 6.8% and 93.2 %, respectively (Figure S6). The percentage of the keto form remaining is somewhat higher than that remaining in the matching diketone **2**. This may be due to an electronic effect by the amide group which is a weaker EWG than its ketone counterpart. As mentioned in the article, the ketone group in bioactive compounds holding the difluorostatone moiety has been shown to participate in serine protease inhibition by nucleophilic attack of the serine-OH on the electrophilic carbonyl carbon to form a hemiketal tetrahedral intermediate.**Error! Bookmark not defined.** In addition, the hydrophobic nature of the pocket in enzymes may change the amount of keto-form present. Indeed, in the more hydrophobic phase of octanol saturated with water, the percentage of the ketone species (36.6%), was much higher than in water even though, in addition to the hemiketal **3-HK**, gem-diol form **3-D** was also formed and observed by ^{19}F-NMR (Figure S6).

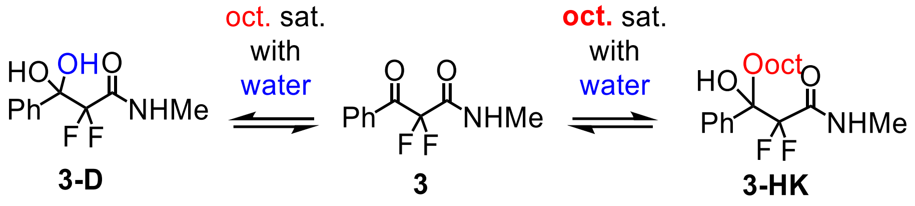

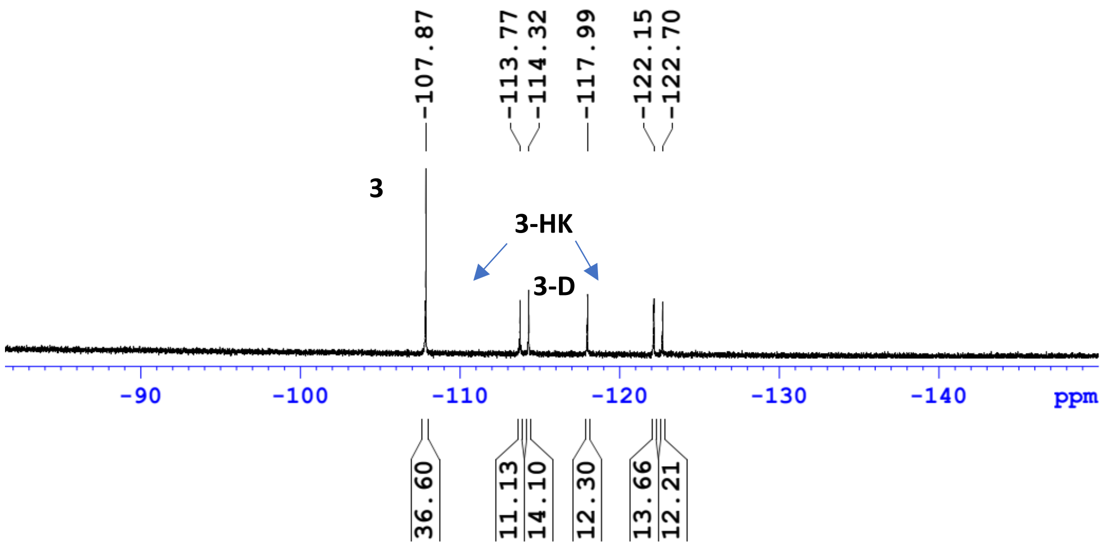

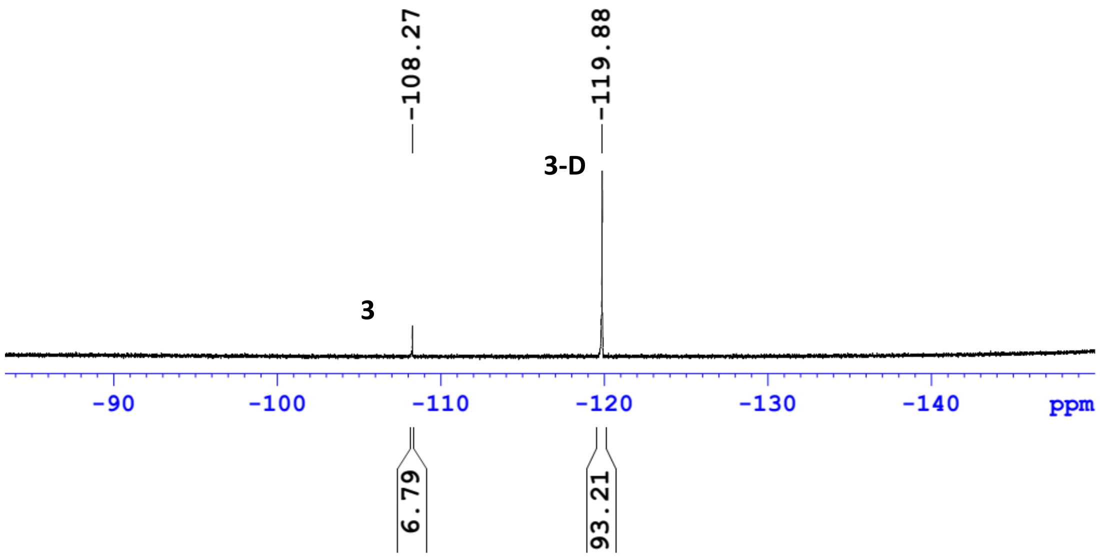

Figure S6. ^{19}F-NMR spectra of diketone **3** in octanol saturated with water (top) and in water (bottom).

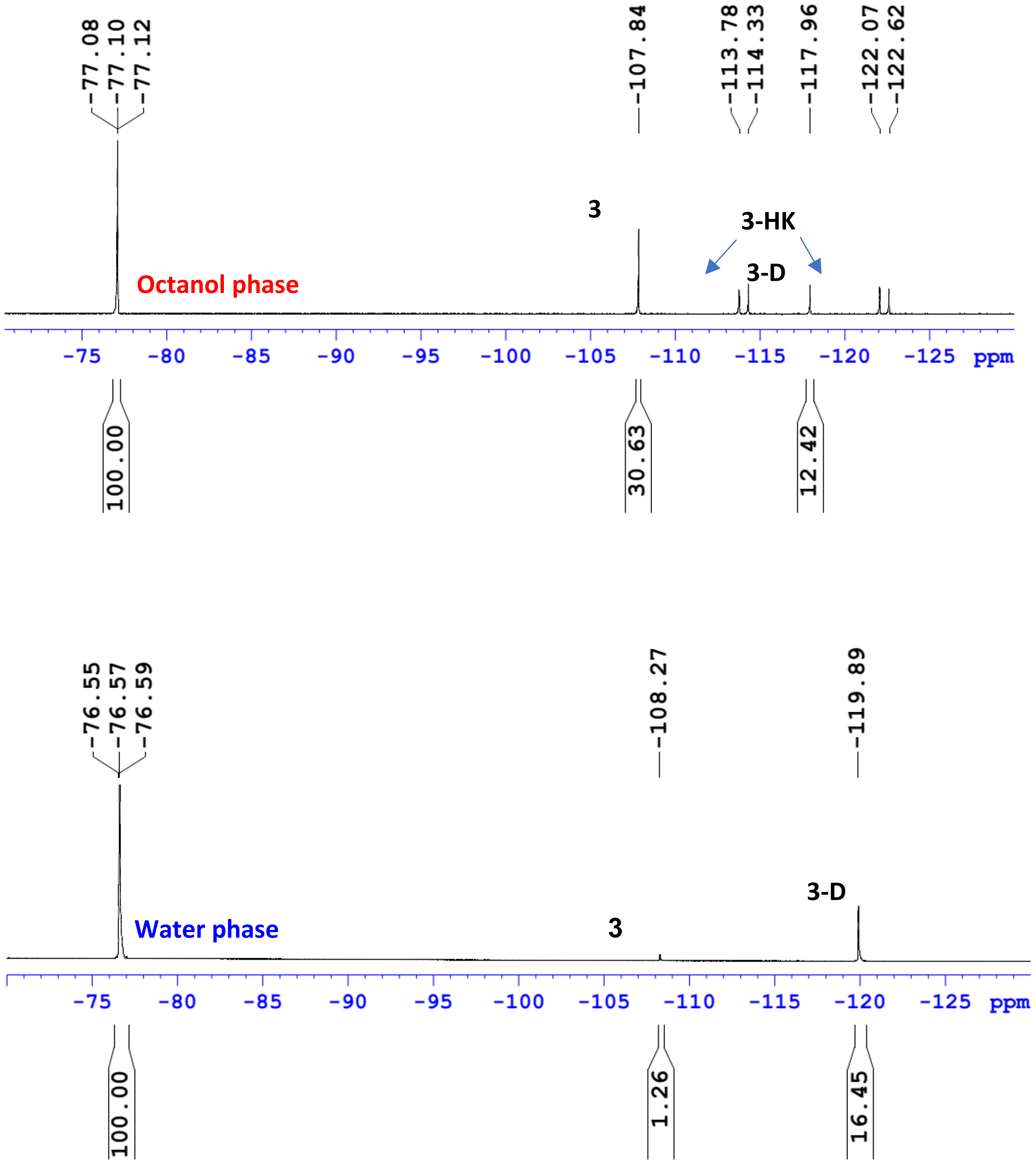

Figure S7. ^{19}F-NMR spectra of diketone **3** in the octanol phase (top) and in the water phase (bottom) after a stir-flask experiment.

.

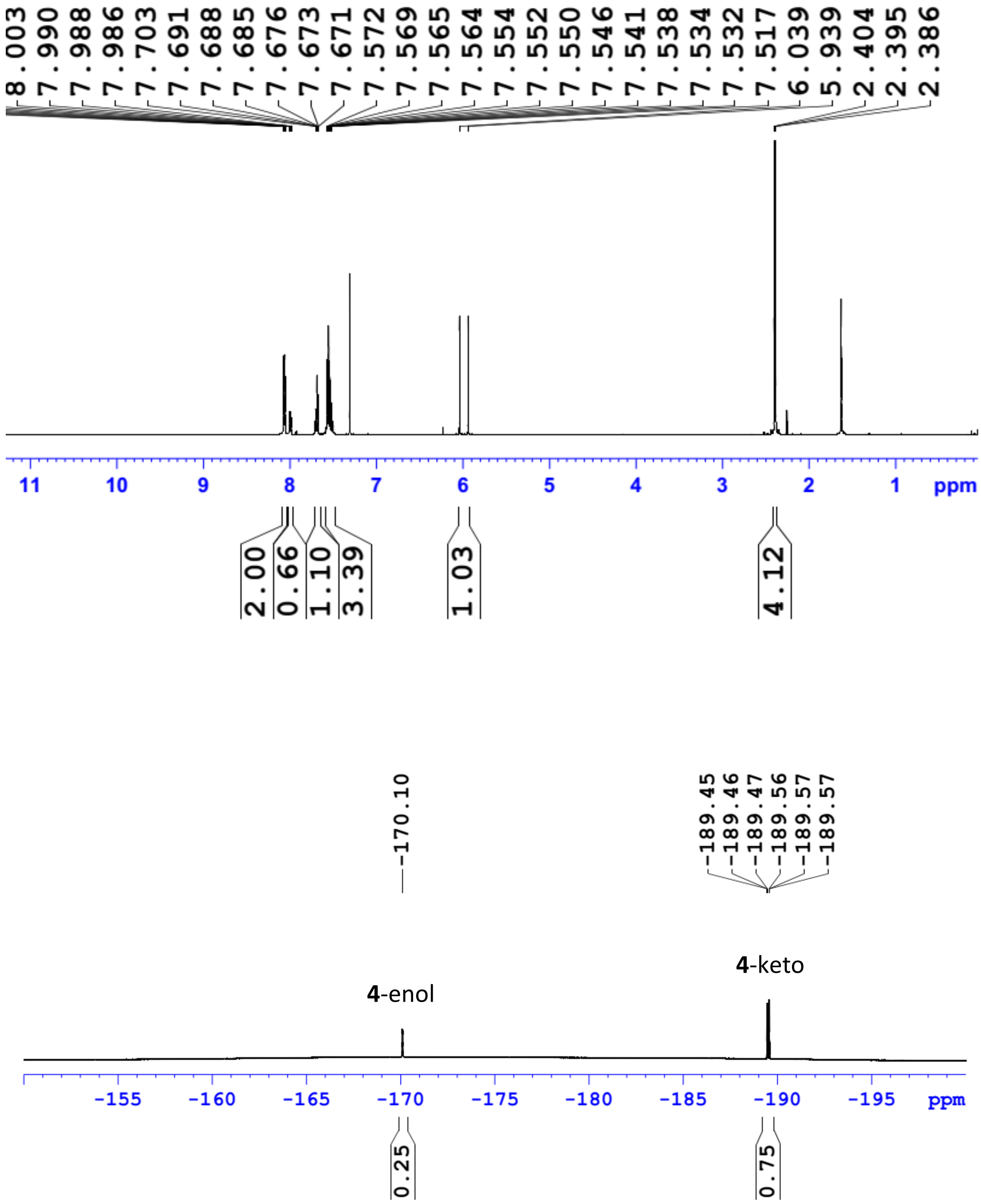

Figure S8. ^{1}H-NMR (top) and ^{19}F-NMR (bottom) spectra of diketone **4** in the $CDCl_3$. The ratio between the keto form and the enol form is 3:1.

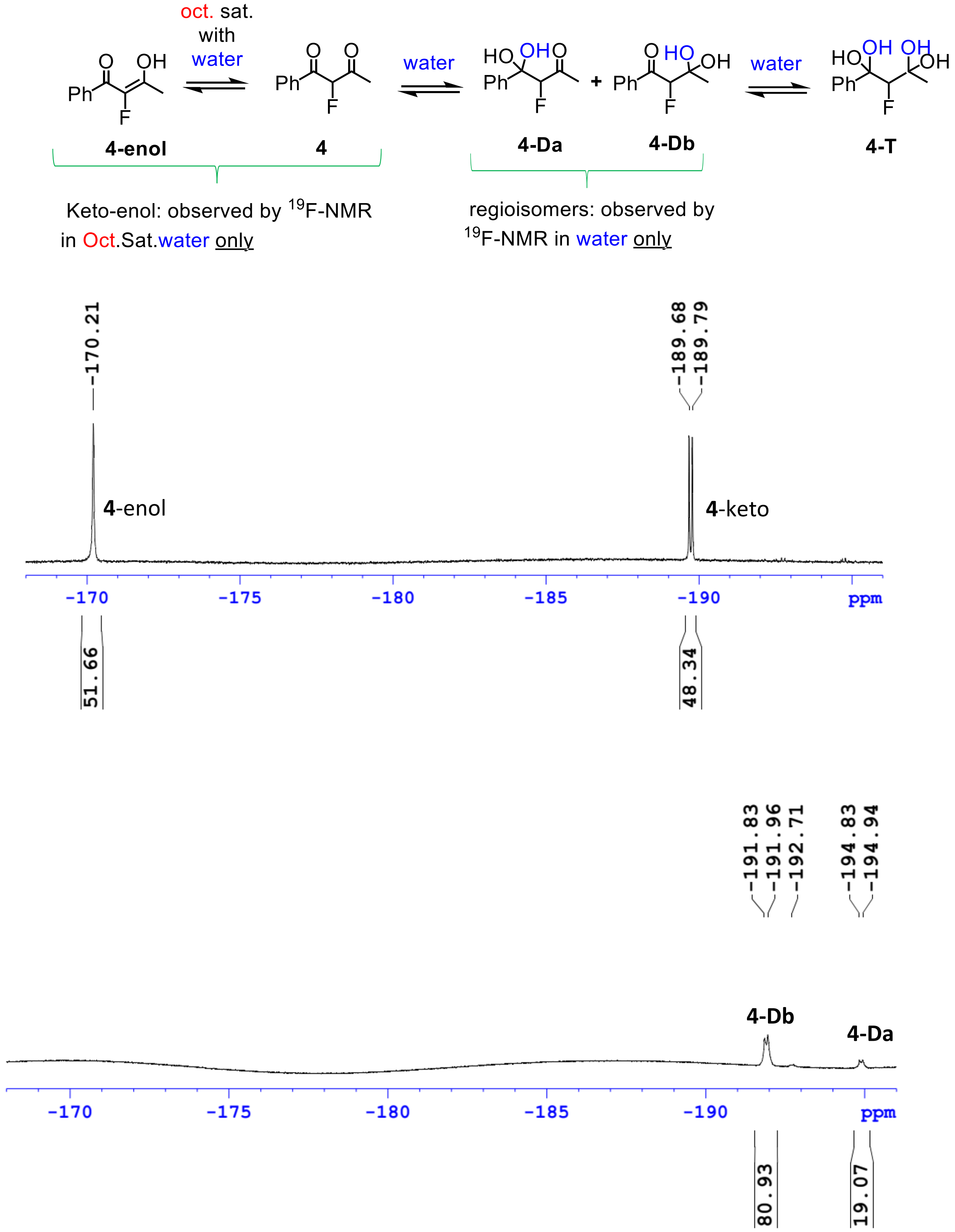

Figure S9. ^{19}F-NMR spectra of diketone **4** in octanol saturated with water (top) and in water (bottom). The assignment of **4-Da** and **4-Db** was based on the assignments of diketones **1** and **2**.

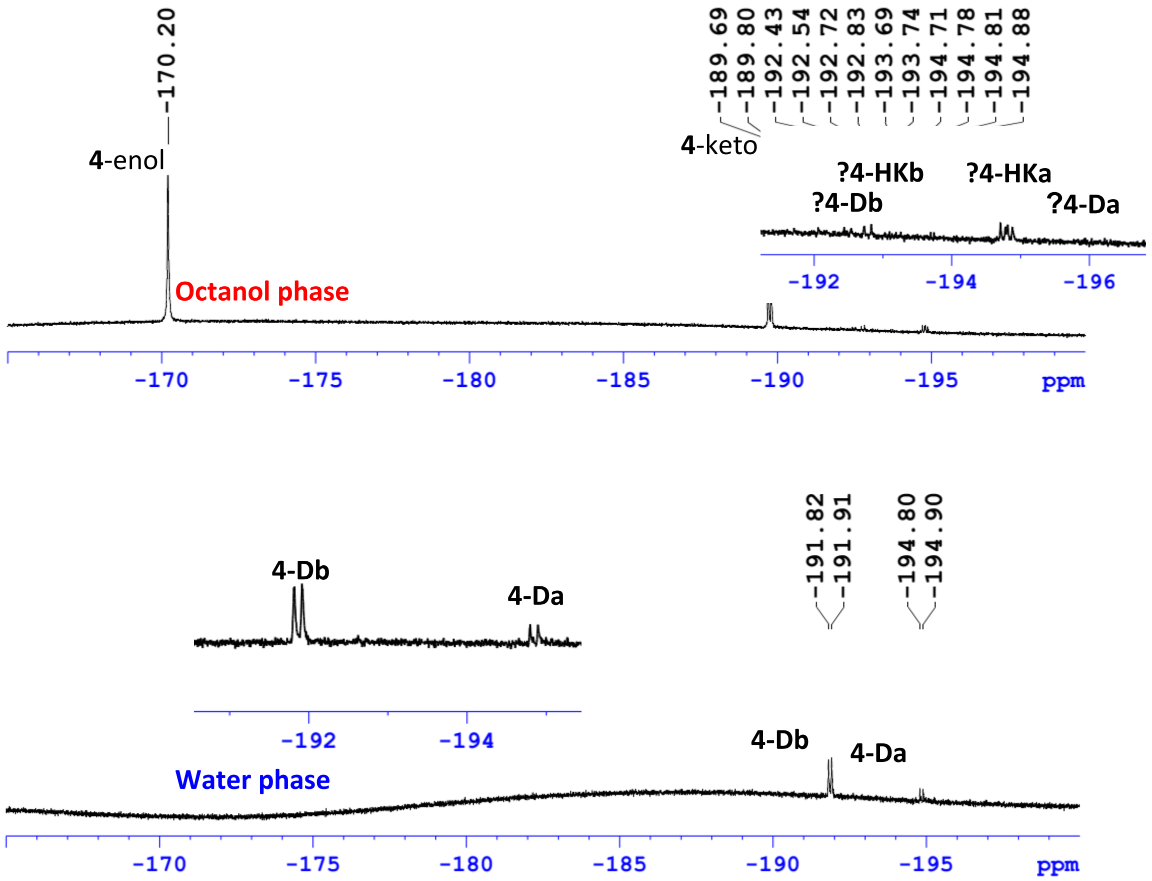

Figure S10. ^{19}F-NMR spectra of diketone **4** in the octanol phase (top) and in the water phase (bottom) after a stir-flask experiment.

.

Explanation of the signal assignments for 5 and 6 and their hydrate and ketal forms.

In the aqueous solution of trifluoromethyl diketone **5**, both **5-enol** and **5-D** were observed (Figure S12), but no **5-keto** could be detected. Similarly, in the solution of octanol saturated with water only the **5-enol** was observed, but not **5-keto**. In addition, contrary to **4**, the corresponding hemiketal **5-HK** and diol **5-D** were also detected in this system, enabling specific log *P* determination of both **5-enol** and **5-D** (vide infra next section). A similar behavior was observed for the difluoromethyl diketone **6**. In water, both **6-enol** and its related diol form **6-D** were observed but not the **6-keto** form (Figure S15), similarly, in octanol saturated with water, **6-enol** was detected, together with the appropriate hemiketal **6-HK** and diol **6-D**, but not the **6-keto** form.

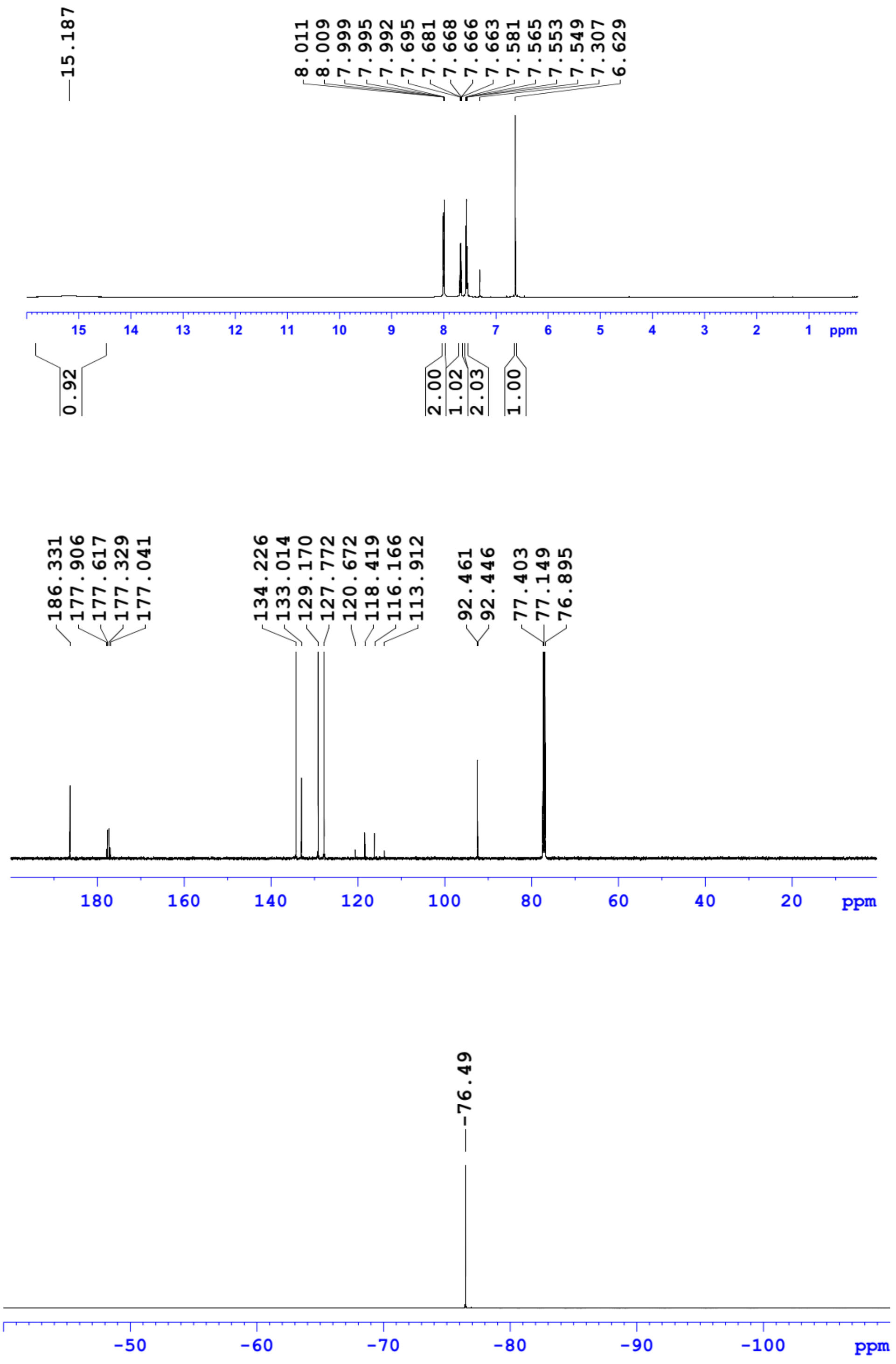

Figure S11. ^{1}H-NMR (top), ^{13}C-NMR (middle) and ^{19}F-NMR (bottom) spectra of diketone **5** in the $CDCl_3$. The assignment of **5** in the enol form is based on the proton spectrum (one vinyl proton at 6.63 ppm).

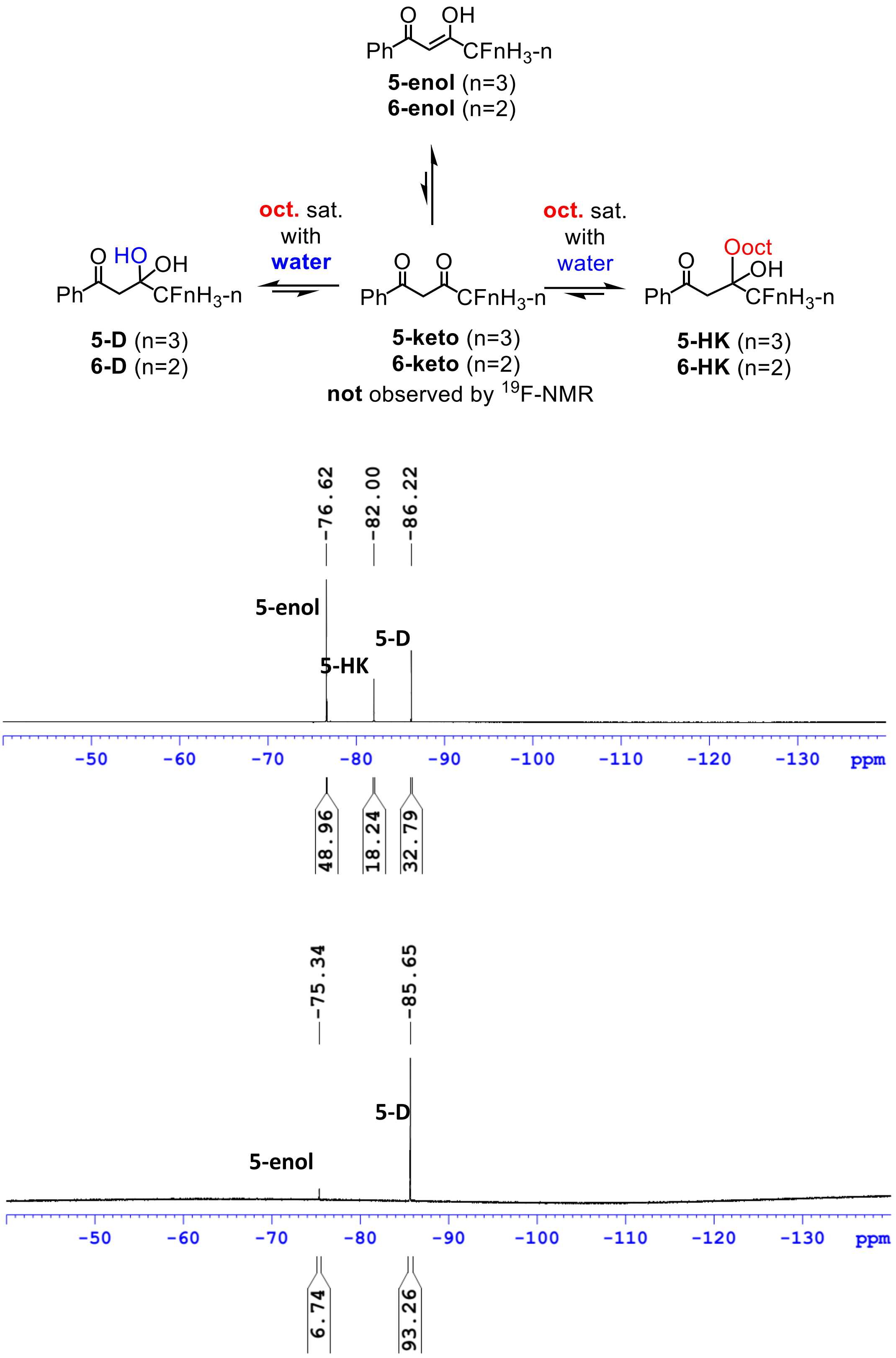

Figure S12. ^{19}F-NMR spectra of **5-enol** in octanol saturated with water (top) and in water (bottom).

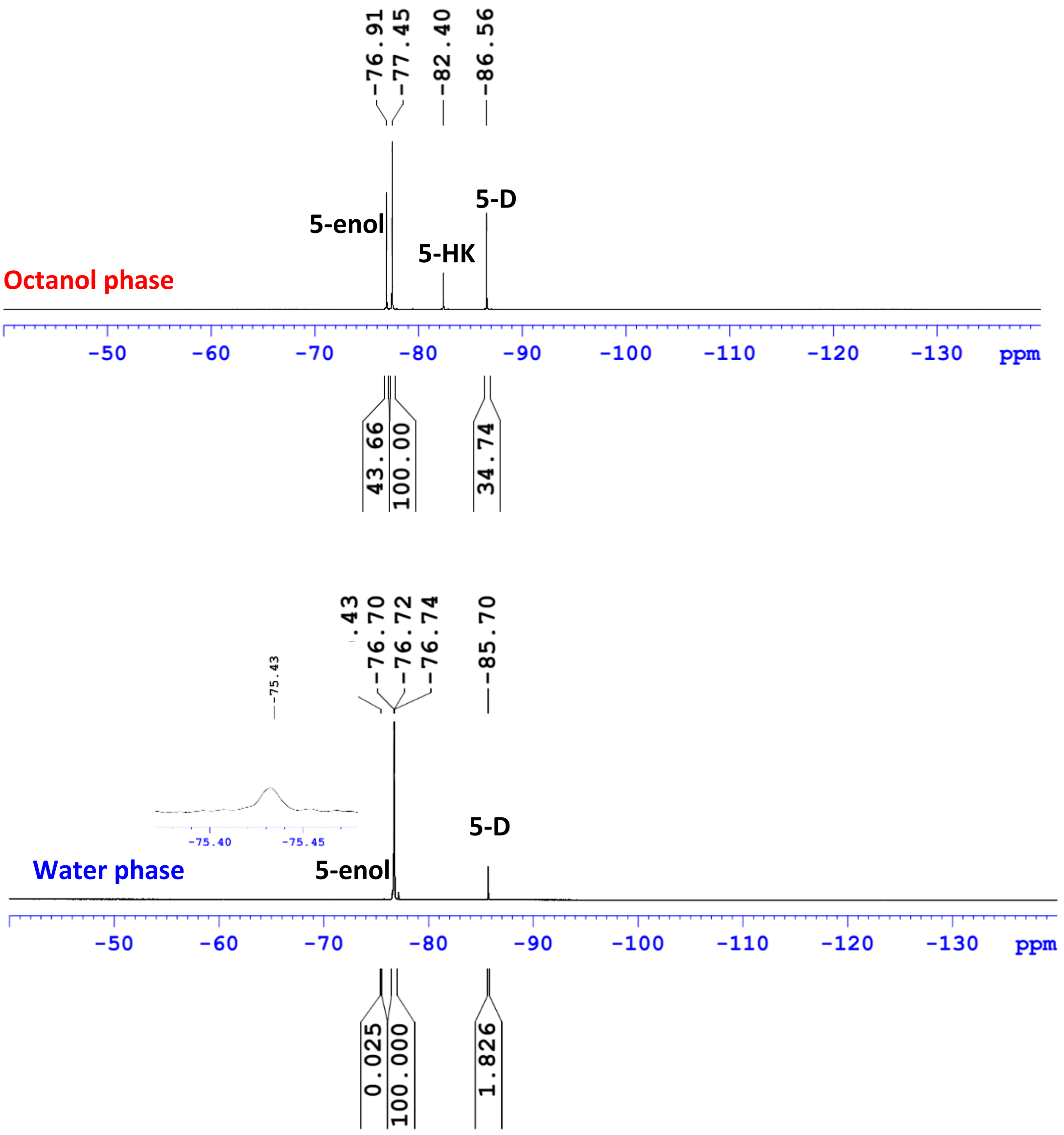

Figure S13. ^{19}F-NMR spectra of **5-enol** in the octanol phase (top) and in the water phase (bottom) after a stir-flask experiment.

.

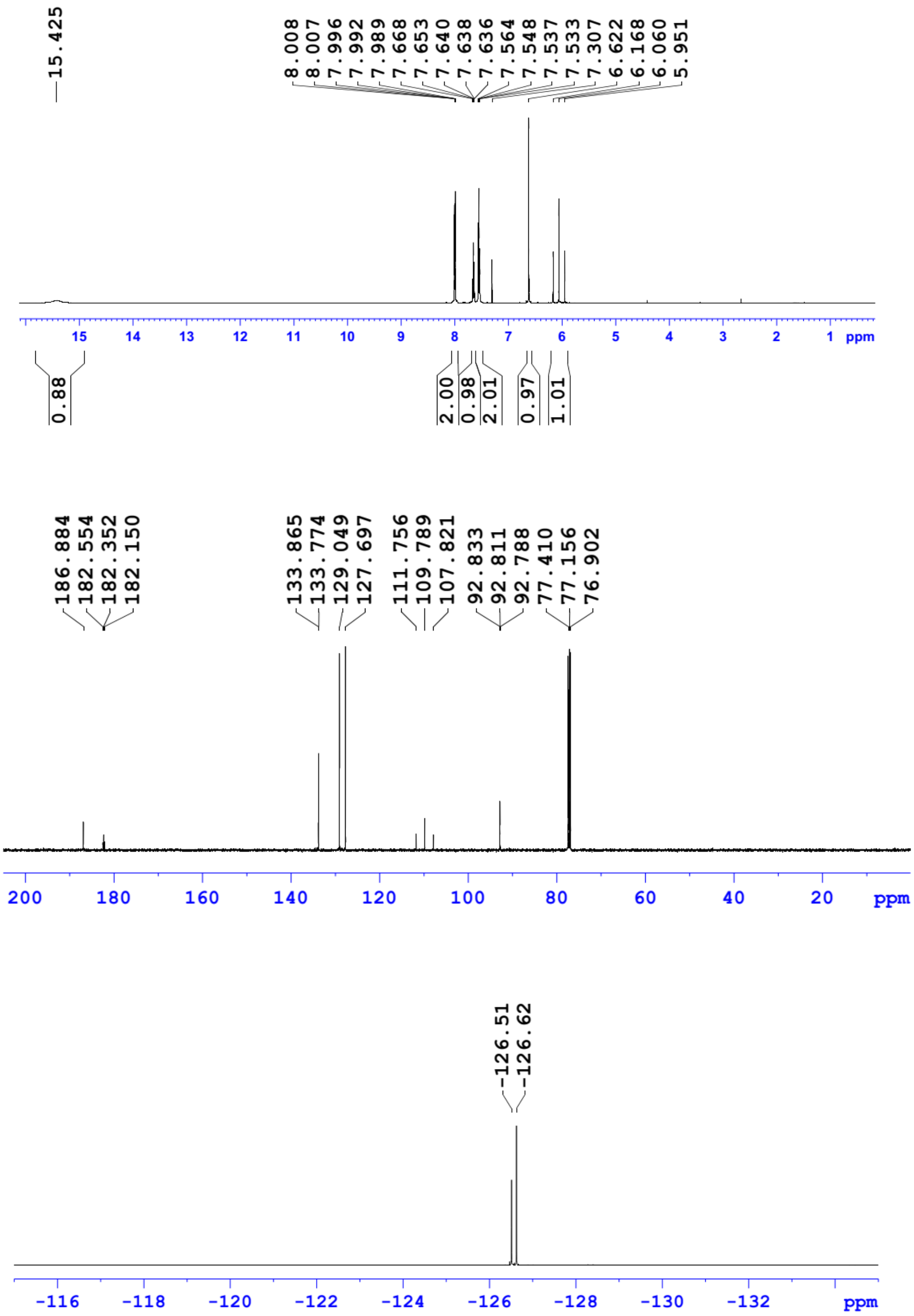

Figure S14. ^{1}H-NMR (top), ^{13}C-NMR (middle) and ^{19}F-NMR (bottom) spectra of diketone **6** in the $CDCl_3$. The assignment of **6** in the enol form is based on the proton spectrum (one vinyl proton at 6.62 ppm).

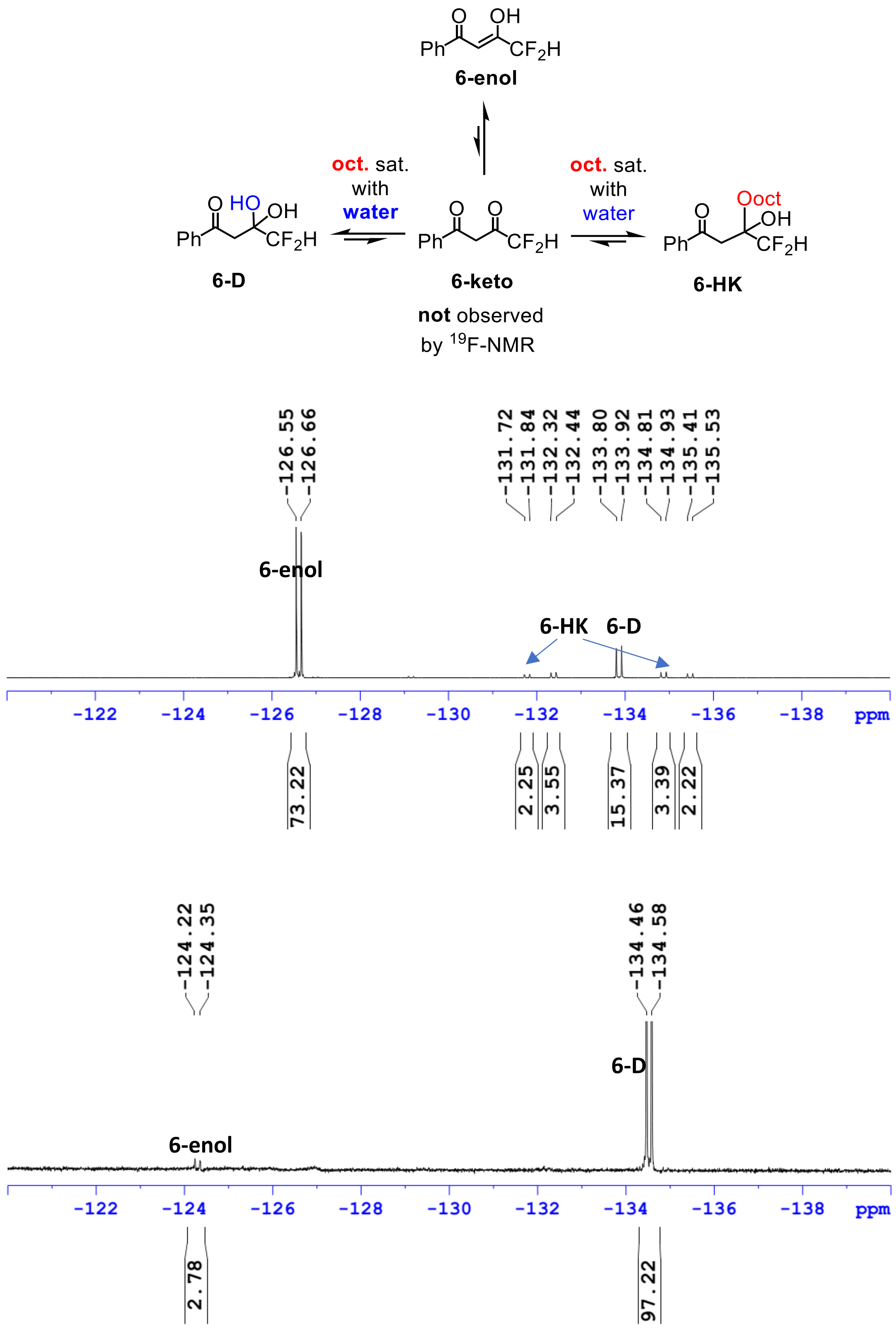

Figure S15. ^{19}F-NMR spectra of **6-enol** in octanol saturated with water (top) and in water (bottom).

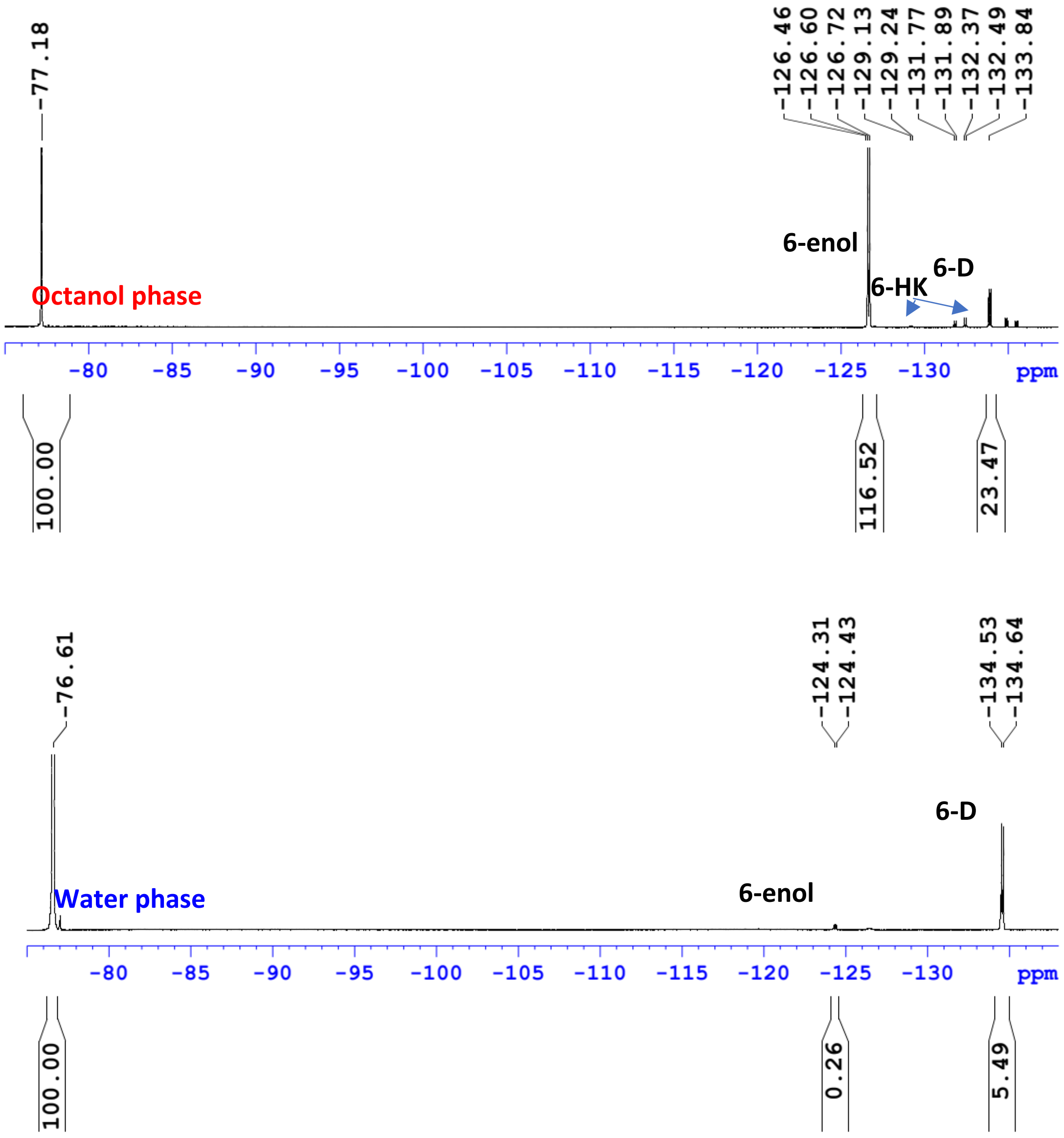

Figure S16. ^{19}F-NMR spectra of **6-enol** in the octanol phase (top) and in the water phase (bottom) after a stir-flask experiment.

.

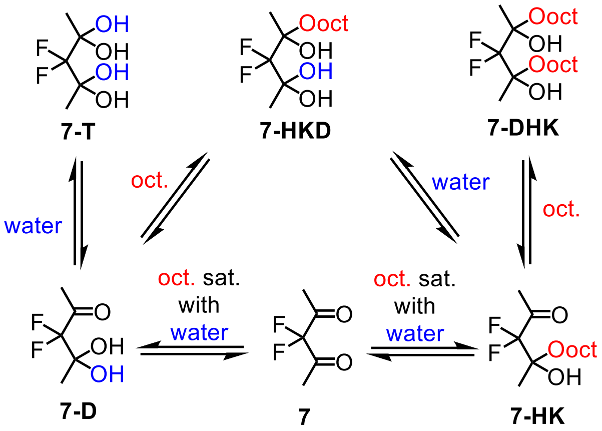

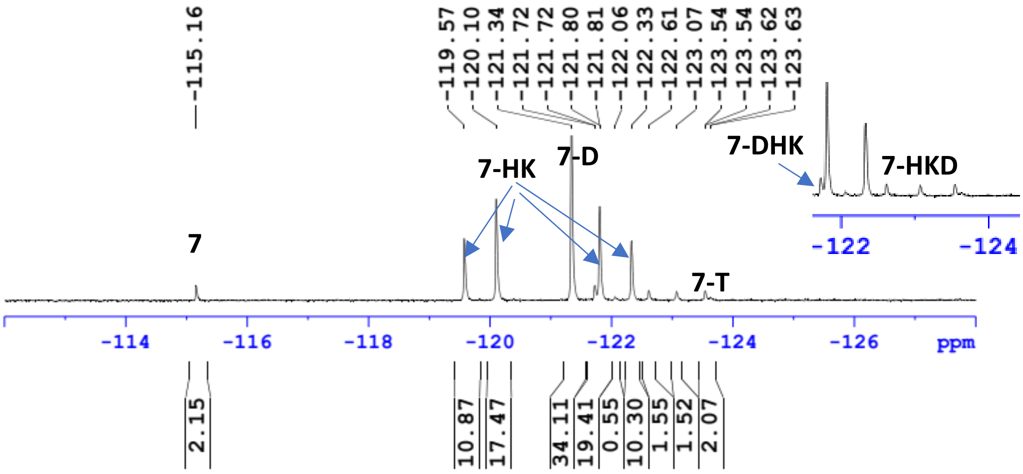

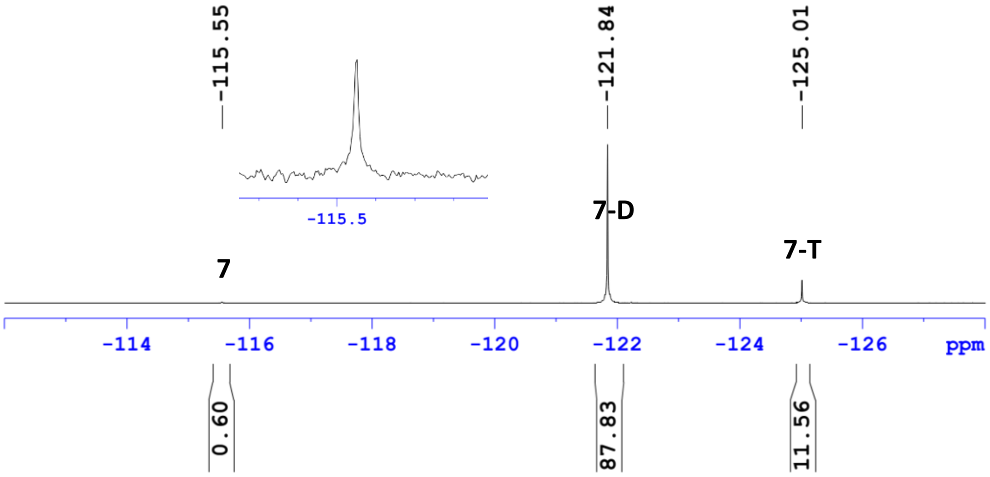

Figure S17. ^{19}F-NMR spectra of **7** in octanol saturated with water (top) and in water (bottom).

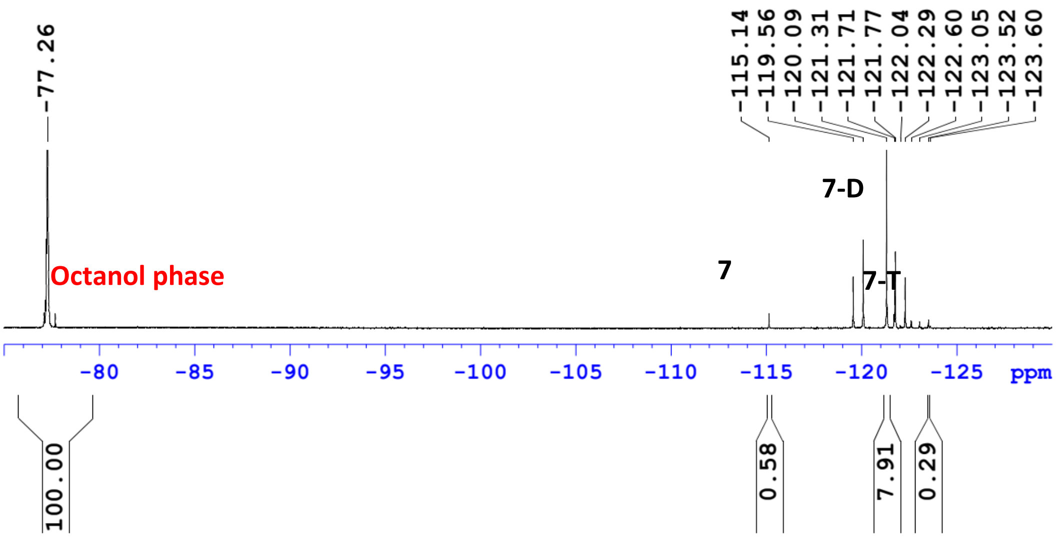

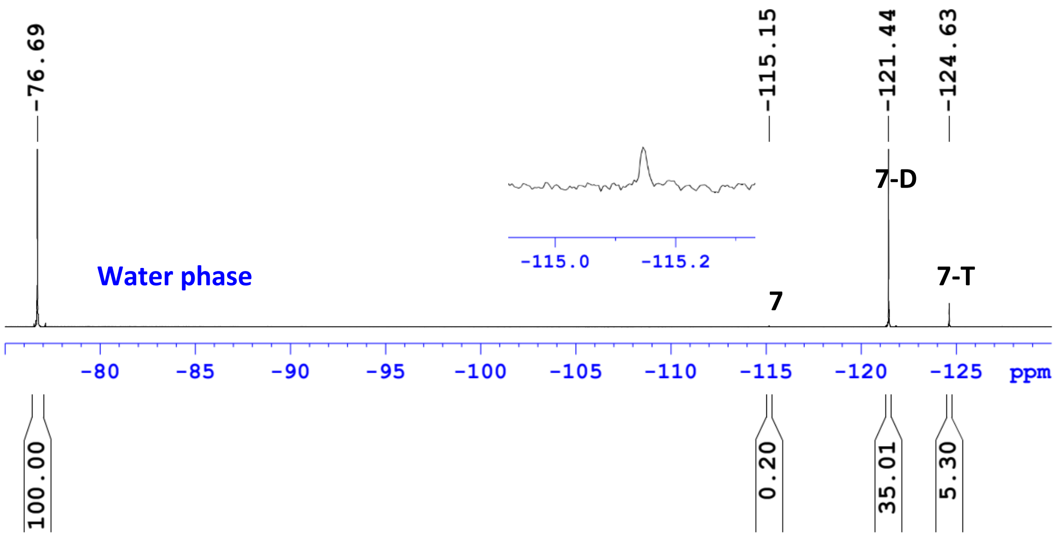

Figure S18. ^{19}F-NMR spectra of **7** in the octanol phase (top) and in the water phase (bottom) after a stir-flask experiment

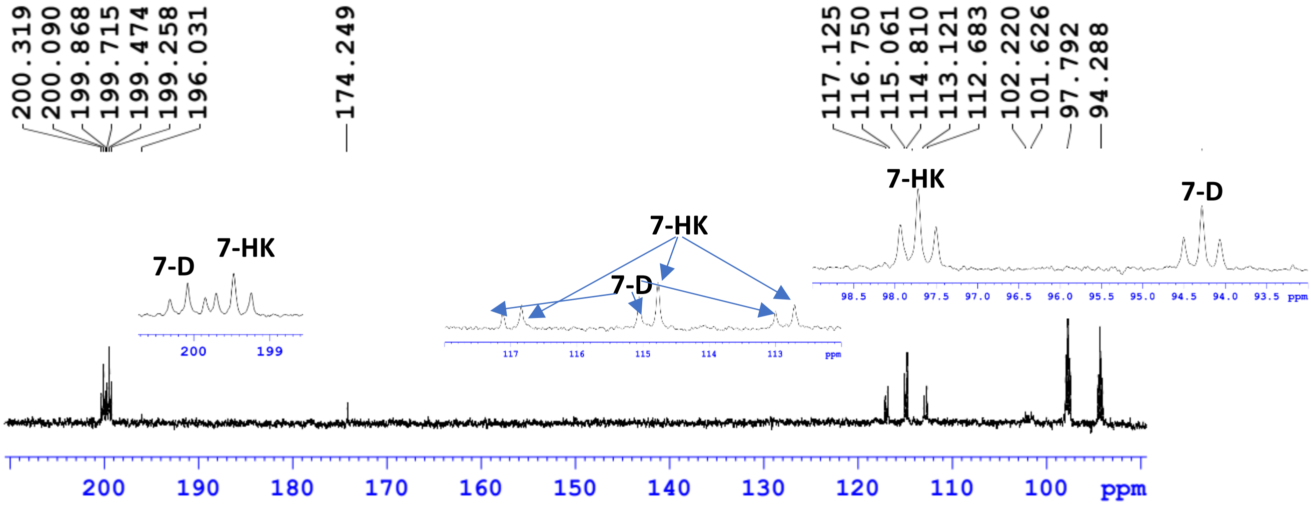

Figure S19. ^{13}C-NMR spectra of diketone **7** in octanol saturated with water.

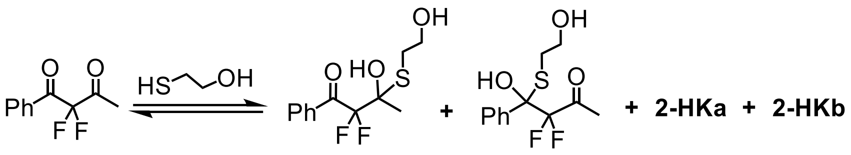

2 (4.1 %) **8** (51.1 %) **9** (40.8 %) 0.9 % 3.2 %

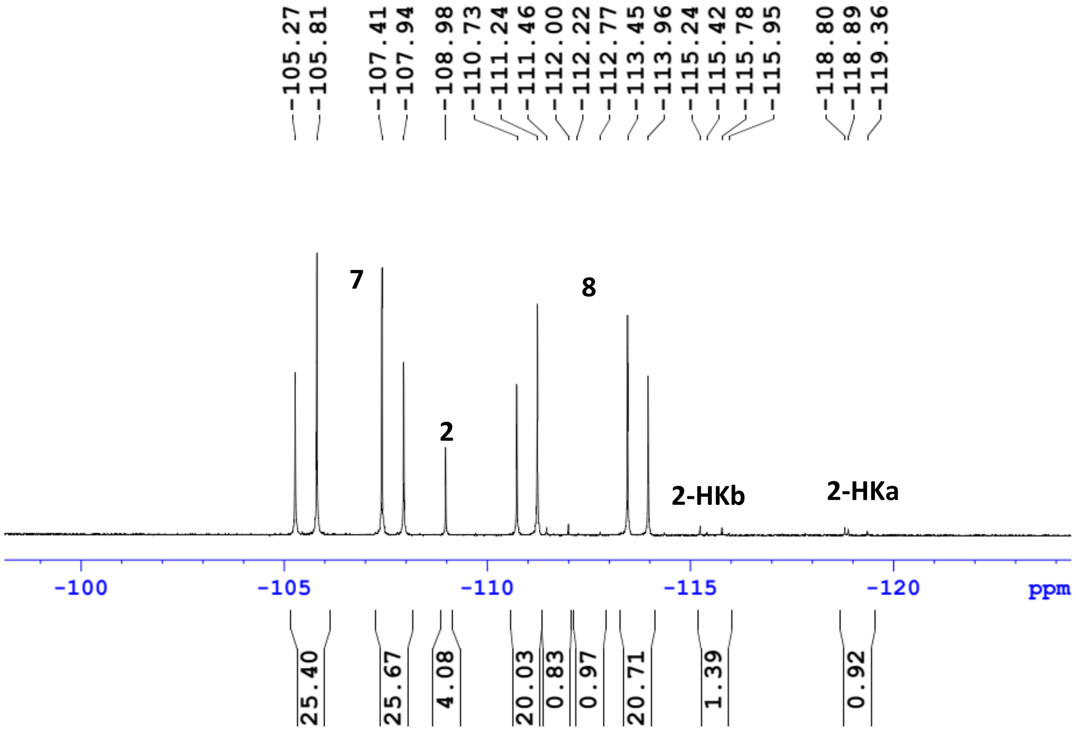

Figure S20. ^{19}F-NMR spectrum of diketone **2** in 2-mercaptoethanol.
